# Supplementary material for: Perioperative haemodynamic therapy for major gastrointestinal surgery: the effect of a Bayesian approach to interpreting the findings of a randomised controlled trial
Source: BMJ Open. 2019 Mar 7;9(3):e024256. doi: 10.1136/bmjopen-2018-024256 (PMC6430028; doi:10.1136/bmjopen-2018-024256)
Supplement: Supplementary data [file bmjopen-2018-024256supp001.pdf]

Supplementary Material

Algorithms settings and code for Bayesian analysis

The Bayesian analysis was conducted in R version 3.4.1 using the *rjags* package [1], which provides an interface to the JAGS library (JAGS version 4.3.0) to perform Bayesian data analysis. JAGS (Just Another Gibbs Sampler) was derived from BUGS (Bayesian analysis Using Gibbs Sampling; see [2] for details on the BUGS project) and uses Markov Chain Monte Carlo (MCMC) to generate samples from the posterior distribution so that inference on the parameters of interest may be obtained. The posterior distribution was obtained using a sample of 200 000 estimates of the primary outcome probability for each arm (used 3 chains each of length 66667, with a burn-in period of 1000 iterations and 500 iterations were used to adapt/tune the samplers). Model convergence was checked using trace plots, density plots, and the Gelman-Rubin diagnostic [3]. The MCMC code is presented below in “R code 1”.

Since we used Beta distributions for the priors, and have binary data that is modelled by a binomial distribution, we are able to obtain an algebraic expression to calculate the posterior distribution. The Beta distribution prior is a *conjugate prior* for the binomial distribution, which means that the posterior distribution is in the same probability distribution family as the prior distribution (Beta distribution). We can update the Beta distribution parameters using the data from the trial:

$$Beta(\alpha_t + y_t, \beta_t + N_t - y_t)$$

where $\mathrm{Beta}(\alpha_{t,}, \beta_t)$ is the prior for treatment arm *t;* y_t are the number of complications/deaths at 30 days for arm *t*; and N_t are the number of patients randomised to arm *t*. We can simulate samples from this distribution for each arm (simulating from the posterior) and examine the relative risks (RR) and absolute risk differences. This code is presented in “R code 2”.

R code 1. Code for posterior simulations using an MCMC algorithm

```
# OPTIMISE trial in a Bayesian framework
# JAMA. 2014;311(21):2181-2190. doi:10.1001/jama.2014.5305


remove(list=ls())


#specify your working directory
setwd("H:/Documents/optimise i")



# Primary outcome: composite of 30-day moderate or major complications and mortality
N1 <- 366
y1 <- 134
```

```
N2 <- 364

y2 <- 158

# N1 is total number in the Cardiac Output–Guided Hemodynamic Therapy Algorithm (intervention) group

# y1 is number with the outcome in the Cardiac Output–Guided Hemodynamic Therapy Algorithm (intervention) group

# N2 is total number in usual care (control) group

# y2 is number with the outcome in usual care (control) group


# Risk ratio

(y1/N1)/(y2/N2)


#frequentist analysis

library(epitools)

riskratio(c(N1-y1, y1, N2-y2, y2), rev="rows", method="boot", replicates=100000)


# Using standard frequentist approach

# Risk ratio (bootstrapped 95% confidence intervals) = 0.84 (0.70-1.01)

# p=0.07 (Fisher exact p-value)



#-------------------------------------------------------------------------------


# Bayesian analysis of same ratio

# Base script from Kruschke, J. K. (2011). Doing Bayesian Data Analysis, Academic Press / Elsevier.


#load required packages (install first if necessary)

require(rjags)

library(BEST)



# The model will be specified with uninformative prior distributions (beta(1,1,).


# Plot prior beta(1,1)

par(mfrow=c(1,1))

plot(seq(0, 1, length.out=100), dbeta(seq(0, 1, length.out=100), 1,1),

                    type="l", xlab="Proportion",

                    ylab="Probability",
```

```
                    main="OPTIMISE Composite Primary Outcome Prior distribution",
                    frame=FALSE, col="red", oma=c(6,6,6,6))
legend("topright", legend="beta(1,1)", lty=1, col="red", inset=0)

#------------------------------------------------------------------------------


# Specify the MODEL.
modelString = "
# JAGS model specification begins here...
model {
# Likelihood. Each complication/death is Bernoulli.
for ( i in 1 : N1 ) { y1[i] ~ dbern( theta1 ) }
for ( i in 1 : N2 ) { y2[i] ~ dbern( theta2 ) }
# Prior. Independent beta distributions. Change this for informative prior
theta1 ~ dbeta( 1,1) #intervention
theta2 ~ dbeta( 1,1) #control
}
# ... end JAGS model specification
" # close quote for modelstring

# Write the modelString to a file, using R commands:
writeLines(modelString,con="H:/Documents/optimise i/model.txt")



#------------------------------------------------------------------------------
# THE DATA.

# Specify the data in a form that is compatible with JAGS model, as a list:
dataList =          list(
          N1 = N1 ,
          y1 = c(rep(1, y1), rep(0, N1-y1)),
          N2 = N2 ,
          y2 = c(rep(1, y2), rep(0, N2-y2))
)

#------------------------------------------------------------------------------
```

```
# INTIALIZE THE CHAIN.

# Can be done automatically in jags.model() by commenting out inits argument.
# Otherwise could be established as:
# initsList = list( theta1 = sum(dataList$y1)/length(dataList$y1) ,
#                   theta2 = sum(dataList$y2)/length(dataList$y2) )

#------------------------------------------------------------------------------
# RUN THE CHAINS.

#specify the MCMC parameters
parameters = c( "theta1" , "theta2" )     # The parameter(s) to be monitored.
adaptSteps = 500              # Number of steps to "tune" the samplers.
burnInSteps = 1000            # Number of steps to "burn-in" the samplers.
nChains = 3                   # Number of chains to run.
numSavedSteps=200000          # Total number of steps in chains to save.
thinSteps=1                   # Number of steps to "thin" (1=keep every step).
nIter = ceiling( ( numSavedSteps * thinSteps ) / nChains ) # Steps per chain.



# Create, initialize, and adapt the model:
jagsModel = jags.model( "H:/Documents/optimise i/model.txt" , data=dataList , # inits=initsList ,
                        n.chains=nChains , n.adapt=adaptSteps )
# Burn-in:
cat( "Burning in the MCMC chain...n" )

#run the MCMC
update( jagsModel , n.iter=burnInSteps )

# The saved MCMC chain:
cat( "Sampling final MCMC chain...n" )
codaSamples = coda.samples( jagsModel , variable.names=parameters ,
                            n.iter=nIter , thin=thinSteps )

# resulting codaSamples object has these indices:
```

```
#   codaSamples[[ chainIdx ]][ stepIdx , paramIdx ]



#check MCMC has converged

plot(codaSamples)

summary(window(codaSamples, start = burnInSteps))

# Gelman-Rubin diagostic - factor of 1 means that between chain variance and within chain variance are equal. Values <=1.1 suggest convergence

gelman.diag(codaSamples)

#Check whether the low chain reduction is stable.

gelman.plot(codaSamples)

#Burn-in appear sufficient (choose the iteration after which the lines stay below 1.1)



#------------------------------------------------------------------------------

# EXAMINE THE RESULTS.


# Convert coda-object codaSamples to matrix object for easier handling.

# This concatenates the different chains into one long chain.

# Result is mcmcChain[ stepIdx , paramIdx ]

mcmcChain = as.matrix( codaSamples )



theta1Sample = mcmcChain[,"theta1"] # Put sampled values in a vector - INTERVENTION ARM.

theta2Sample = mcmcChain[,"theta2"] # Put sampled values in a vector - CONTROL ARM




# Plot a histogram of the posterior differences of theta values.

thetaRR = theta1Sample / theta2Sample # Relative risk

thetaDiff = theta1Sample - theta2Sample # Absolute risk difference


# 2x1 plots

par(mfrow=c(2,1))


# plot posterior of RR

par(mar=c(4, 4.1, 4.1, 4))
```

```
plotPost( thetaRR , xlab= expression(paste("Relative risk (", theta[1]/theta[2], ")")) ,
	compVal=1.0, ROPE=c(0.9, 1.1),
	main="OPTIMISE Composite Primary Outcome
Posterior distribution of relative risk", cex.main=1, cex=1, cex.lab=1, xlab=c(0.6,1.4))

#Plot absolute risk difference
plotPost( thetaDiff , xlab=expression(paste("Absolute risk difference (", theta[1]-theta[2], ")")) ,
	compVal=0.0, ROPE=c(-0.05, 0.05),
	main="OPTIMISE Composite Primary Outcome
Posterior distribution of absolute risk difference", cex.main=1, cex=1, cex.lab=1, xlab=c(-0.25, 0.15))




#------------------------------------------------------------------------------

# Use posterior prediction to determine proportion of cases in which
# using the intervention would result in no complication/death
# while not using the intervention would result in complication death

chainLength = length( theta1Sample )

# Create matrix to hold results of simulated patients:
yPred = matrix( NA , nrow=2 , ncol=chainLength )

# For each step in chain, use posterior prediction to determine outcome
for ( stepIdx in 1:chainLength ) { # step through the chain
	# Probability for complication/death for each "patient" in intervention group:
	pDeath1 = theta1Sample[stepIdx]
	# Simulated outcome for each intervention "patient"
	yPred[1,stepIdx] = sample( x=c(0,1), prob=c(1-pDeath1,pDeath1), size=1 )
	# Probability for complication/death for each "patient" in control group:
	pDeath2 = theta2Sample[stepIdx]
	# Simulated outcome for each control "patient"
	yPred[2,stepIdx] = sample( x=c(0,1), prob=c(1-pDeath2,pDeath2), size=1 )
}
```

```
# Now determine the proportion of times that the intervention group has no complication/death
# (y1 == 0) and the control group does have a complication or death (y2 == 1))
(pY1eq0andY2eq1 = sum( yPred[1,]==0 & yPred[2,]==1 ) / chainLength)

#proportion of times intervention has complication/death where control group does not
(pY1eq1andY2eq0 = sum( yPred[1,]==1 & yPred[2,]==0 ) / chainLength)

#proportion of times where neither arm has complication/death
(pY1eq0andY2eq0 = sum( yPred[1,]==0 & yPred[2,]==0 ) / chainLength)

#proportion of times where both arms have complication/death
(pY10eq1andY2eq1 = sum( yPred[1,]==1 & yPred[2,]==1 ) / chainLength)
```

R Code 2. Code for exact posterior calculation

```
remove(list=ls())

###uninformative prior

#prior for control group is beta(a1, b1)
a1=1
b1=1

#prior for treatment group is beta(a2, b2)
a2=1
b2=1

#control event/total is z1/N1
z1=158
N1=364

#treatment event/total is z2/N2.
z2=134
N2=366

#simulate from the posterior for control group. posterior can be calculated exactly as beta(a1+z1, b1+N1-z1)
pc = rbeta(200000, a1+z1, b1+N1-z1)
#simulate from the posterior for intervention group. posterior can be calculated exactly as beta(a2+z2, b2+N2-z2)
pt = rbeta(200000, a2+z2, b2+N2-z2)


thetaRR = pt / pc # Relative risk
thetaDiff = pt - pc # Absolute risk difference

##########################################################################
##########################################################################
#evidence-based prior

#prior for control group is beta(a1, b1)
a1=24.26
b1=31.58


#prior for treatment group is beta(a2, b2)
a2=16.46
b2=49.17

#control event/total is z1/N1
z1=158
N1=364

#treatment event/total is z2/N2.
z2=134
N2=366

#simulate from the posterior for control group. posterior can be calculated exactly as beta(a1+z1, b1+N1-z1)
```

```
pcc = rbeta(200000, a1+z1, b1+N1-z1)
#simulate from the posterior for intervention group. posterior can be calculated exactly as beta(a2+z2, b2+N2-z2)
ptt = rbeta(200000, a2+z2, b2+N2-z2)



thetaRR_ev = ptt / pcc # Relative risk
thetaDiff_ev = ptt - pcc # Absolute risk difference


#####################################################################

#plot posterior distributions

require(rjags)
library(BEST)

# 2x2 plots
par(mfrow=c(2,2))

# plot posterior for uninformative prior, RR
par(mar=c(4, 4.1, 4.1, 4))
plotPost( thetaRR , xlab= expression(paste("          Relative risk (", theta[1]/theta[2], ")")) ,
          compVal=1.0, ROPE=c(0.9, 1.1),
          main="a)", adj=0,cex.main=1, cex=1, cex.lab=1, xlab=c(0.6,1.4))

# plot posterior for uninformative prior, absolute risk difference
plotPost( thetaDiff , xlab=expression(paste("Absolute risk difference (", theta[1]-theta[2], ")")) ,
          compVal=0.0, ROPE=c(-0.05, 0.05),
          main="b)", adj=0,cex.main=1, cex=1, cex.lab=1, xlab=c(-0.25, 0.15))

# plot posterior for evidence-based prior, RR
par(mar=c(4, 4.1, 4.1, 4))
plotPost( thetaRR_ev , xlab= expression(paste("          Relative risk (", theta[1]/theta[2], ")")) ,
          compVal=1.0, ROPE=c(0.9, 1.1),
          main="c)", adj=0,cex.main=1, cex=1, cex.lab=1, xlab=c(0.6,1.4))

# plot posterior for evidence-based prior, absolute risk difference
plotPost( thetaDiff_ev , xlab=expression(paste("Absolute risk difference (", theta[1]-theta[2], ")")) ,
          compVal=0.0, ROPE=c(-0.05, 0.05),
          main="d)", adj=0,cex.main=1, cex=1, cex.lab=1, xlab=c(-0.25, 0.15))
```

Evidence-based Prior

An evidence-based prior was specified using results from a pre-existing meta-analysis ([4,5] – not including the OPTIMISE results) which had information on both treatment arms. There is no clear best approach of how to include historical information into the analysis of a clinical trial. Viele et al. [6] provide a good overview of the available approaches when data for the control arm is available and discuss how to decide to what extent historical data should be incorporated.

The R Bayesian evidence synthesis Tools (RBesT; [7]) were used to perform a Bayesian meta-analysis, which accounts for the uncertainty of the population mean and between-trial heterogeneity (using the *gMAP* function in R (version 3.4.1)). We used this approach as it is a principled and reproducible method of combining data from previous trials. Further refinements could also be included, such as attempting to correct for publication or other biases. The meta-analysis of the control and intervention arms are displayed in Figures S1 and S2, respectively.

For the control arm, the meta-analysis gave a mean estimate of 0.432 (95% HDI: 0.306, 0.563). Using the *betaExpert* function (from the *prevalence* package [8]) in R version 3.4.1 (setting the mean from the meta-analysis to the mode of the beta distribution, and using the 95% HDI) gave a beta(24.26,31.58) distribution. This was used as the prior for the control arm.

For the intervention arm, the meta-analysis gave a mean estimate of 0.243 (95% HDI: 0.148, 0.358). Using the *betaExpert* function [8] in R (setting the mean from the meta-analysis to the mode of the beta distribution, and using the 95% HDI) gave a beta(16.46, 49.17) distribution. This was used as the prior for the intervention arm.

We note that power priors [9] could have been used instead for deriving the evidence-based priors, which incorporate a weight parameter that determines how much information is used from the historical data.

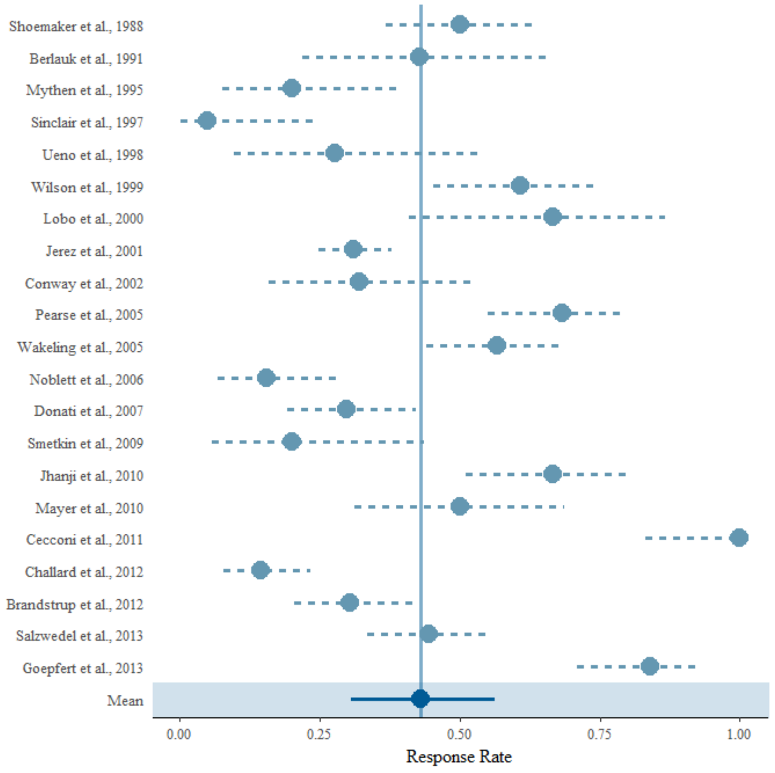

Figure S1.Forest plot of control arm using meta-analysis from Pearse et al. [5]. Dashed lines represent the stratified estimates with 95% HDI. Solid line represents the mean estimate (with 95% HDI))

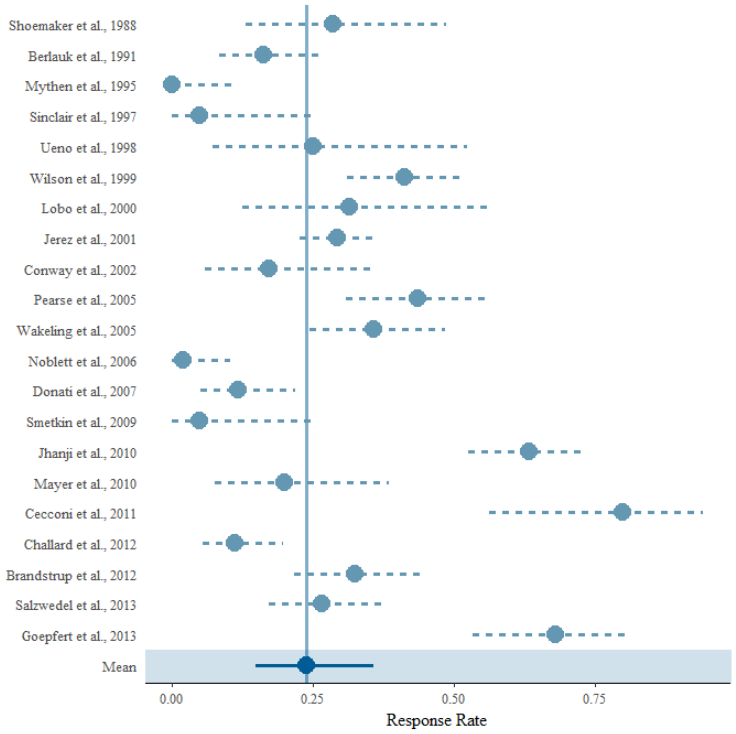

Figure S2. Forest plot of intervention arm using meta-analysis from Pearse et al. [5]. Dashed lines represent the stratified estimates with 95% HDI. Solid line represents the mean estimate (with 95% HDI))

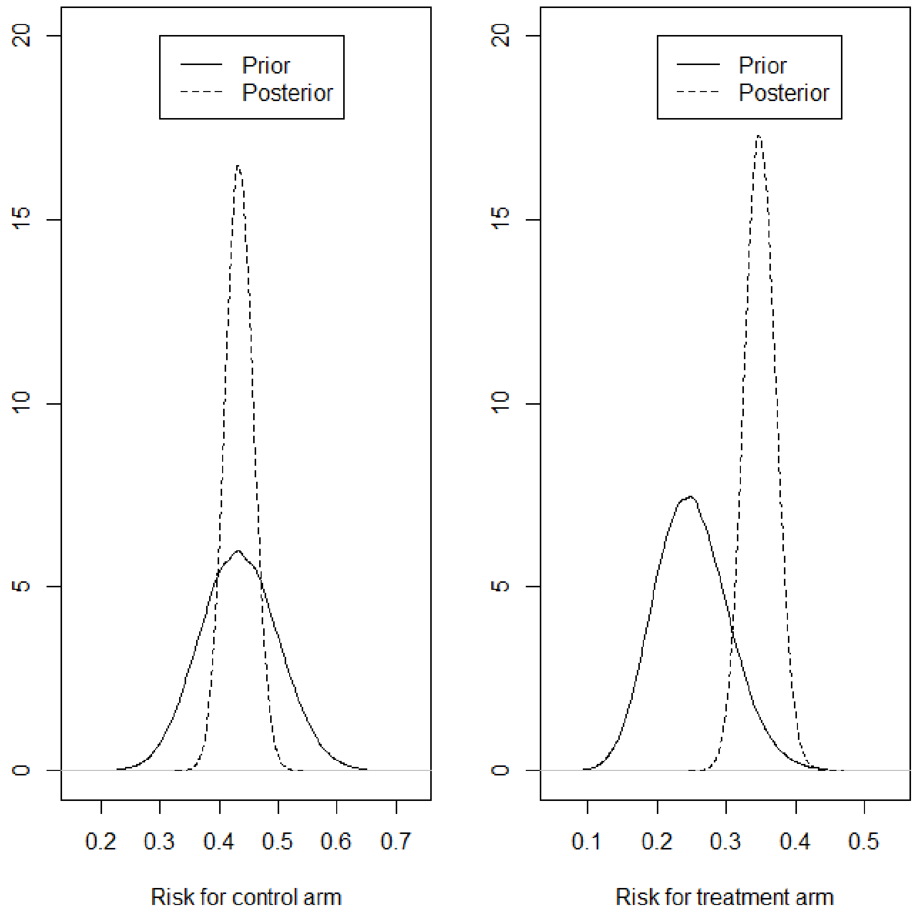

Figure S3. Plot of the evidence-based priors overlaid with the posteriors for each treatment arm

References

[1] Plummer M. JAGS: A program for analysis of Bayesian graphical models using Gibbs sampling. Proceedings of the 3rd International Workshop on Distributed Statistical Computing (DSC 2003). March 2003. p. 20–2.

[2] Lunn D, Spiegelhalter D, Thomas A, Best N. The BUGS project: Evolution, critique and future directions. Stat Med. 2009 October 13; 28(25): 3049-3067

[3] Gelman A, Rubin DB. Inference from Iterative Simulation Using Multiple Sequences. Stat Sci. 1992; 7(4): 457-472.

[4] Grocott MP, Dushianthan A, Hamilton MA, Mythen MG, Harrison D, Rowan K; Optimisation Systematic Review Steering Group. Perioperative increase in global blood flow to explicit defined goals and outcomes following surgery. *Cochrane Database Syst Rev*. 2012 Nov 14;11:CD004082.

[5] Pearse RM, Harrison DA, MacDonald N, Gillies MA, Blunt M, Ackland G, et al. Effect of a Perioperative, Cardiac Output–Guided Hemodynamic Therapy Algorithm on Outcomes

Following Major Gastrointestinal Surgery A Randomized Clinical Trial and Systematic Review. JAMA. 2014 May 19; 311(21): 2181-2190.

[6] Viele K, Berry S, Neuenschwander B, Amzal B, Chen F, Enas N, et al. Use of historical control data for assessing treatment effects in clinical trials. Pharm Stat. 2013 August 5; 13:41-54

[7] Weber S. (2017). RBesT: R Bayesian Evidence Synthesis Tools. R package version 1.2-3. https://CRAN.R-project.org/package=RBesT

[8] Devleesschauwer B, Torgerson P, Charlier J, Levecke B, Praet N, Roelandt S, et al. (2014). prevalence: Tools for prevalence assessment studies. R package version 0.4.0. http://cran.r-project.org/package=prevalence

[9] Chen M-H, Ibrahim JG, Shao Q-M. Power prior distributions for generalized linear models. J Stat Plan Infer. 2000 Mar; 84(1-2):121-137
