# Supplementary material for: Perioperative haemodynamic therapy for major gastrointestinal surgery: the effect of a Bayesian approach to interpreting the findings of a randomised controlled trial
Source: BMJ Open. 2019 Mar 7;9(3):e024256. doi: 10.1136/bmjopen-2018-024256 (PMC6430028; doi:10.1136/bmjopen-2018-024256)
Supplement: Author's manuscript [file bmjopen-2018-024256.draft_revisions.pdf]

BMJ Open is committed to open peer review. As part of this commitment we make the peer review history of every article we publish publicly available.

When an article is published we post the peer reviewers' comments and the authors' responses online. We also post the versions of the paper that were used during peer review. These are the versions that the peer review comments apply to.

The versions of the paper that follow are the versions that were submitted during the peer review process. They are not the versions of record or the final published versions. They should not be cited or distributed as the published version of this manuscript.

BMJ Open is an open access journal and the full, final, typeset and author-corrected version of record of the manuscript is available on our site with no access controls, subscription charges or pay-per-view fees (http://bmjopen.bmj.com).

If you have any questions on BMJ Open's open peer review process please email info.bmjopen@bmj.com

BMJ Open

Perioperative haemodynamic therapy for major gastrointestinal surgery: The effect of a Bayesian approach to interpreting the findings of randomised controlled trials

Journal:	*BMJ Open*
Manuscript ID	bmjopen-2018-024256
Article Type:	Research
Date Submitted by the Author:	17-May-2018

Complete List of Authors:	Ryan, Elizabeth; University of Warwick, Clinical Trials Unit<br>Harrison, Ewen; Univeristy of Edinburgh, Clinical Surgery<br>Pearse, Rupert; Queen Mary University of London, Barts and the London School of Medicine & Dentistry<br>Gates, Simon; University of Birmingham, Cancer Research UK Clinical Trials Unit; University of Warwick, Clinical Trials Unit

Keywords:	Bayesian analysis, randomised controlled trial, SURGERY, peri-operative care

SCHOLARONE™
Manuscripts

Perioperative haemodynamic therapy for major gastrointestinal surgery: The effect of a Bayesian approach to interpreting the findings of randomised controlled trials

Elizabeth G. Ryan*[†], Ewen M. Harrison*, Rupert M. Pearse, and Simon Gates

***joint first authors**

[†]Corresponding author
Dr Elizabeth G. Ryan
Clinical Trials Unit, Warwick Medical School, University of Warwick, Coventry, United Kingdom CV4 7AL
phone: (+44) 024 765 74653

Email addresses:
EGR: L.Ryan.4@warwick.ac.uk
EMH: mail@ewenharrison.com
RMP: r.pearse@qmul.ac.uk
SG: S.Gates@bham.ac.uk

For peer review only

Abstract

Objective: The traditional approach of null hypothesis testing dominates the design and analysis of randomised controlled trials. This study aimed to demonstrate how a simple Bayesian analysis could have been used to analyse the OPTIMISE trial to obtain more clinically interpretable results.

Design, setting, participants, and interventions: The OPTIMISE trial was a pragmatic, multicentre, observed-blinded, randomised controlled trial of 734 high-risk patients undergoing major gastrointestinal surgery in 17 acute care hospitals in the United Kingdom. Patients were randomly allocated to a cardiac output-guided hemodynamic therapy algorithm for intravenous fluid and inotropic drug administration during and in the 6 hours following surgery (n=368) or to standard care (n=366). The primary outcome was a composite of predefined 30-day moderate or major complications and mortality.

Methods: We repeated the primary outcome analysis of the OPTIMISE trial using Bayesian statistical methods to calculate the probability that the intervention was superior, and the probability that a clinically relevant difference existed.

Results: Although OPTIMISE was not powered to detect a statistically significant difference between the treatment arms for the observed effect size (RR=0.84, 95% CI: 0.70, 1.01; p=0.07), by using Bayesian analyses we were able to demonstrate that there was a 97-99.5% probability that the intervention was superior to the control.

Conclusions: The use of a Bayesian analytical approach provided a different interpretation of the findings of the OPTIMISE trial (compared to the original frequentist analysis), and suggested patient

benefit from the intervention. Bayesian analyses can produce results that are more easily interpretable and relevant to clinicians and policy makers.

Trial registration: isrctn.org Identifier: ISRCTN04386758

Keywords: Bayesian analysis; randomised controlled trial; surgery; peri-operative care

Word count: 2262

Article Summary

Strengths and limitations

- The use of a Bayesian analytical approach provided a different interpretation of the findings of the OPTIMISE trial (compared to the original frequentist analysis).
- The Bayesian approach avoids the dichotomisation of results that occur in significance testing.
- Bayesian analyses can produce results that are more easily interpretable and relevant to clinicians and policy makers.
- The analysis only used simple Bayesian methods and more sophistication can be included, such as adjusting for covariates.

Funding: EGR and SG were supported by a Medical Research Council (MRC) Methodology Research Grant (Grant number: MR/N028287/1).

Competing Interests: RMP holds research grants, and has given lectures and/or performed consultancy work for Nestle Health Sciences, BBraun, Medtronic, Glaxo Smithkline, Intersurgical, and Edwards Lifesciences, and is a member of the Associate editorial board of the British Journal of Anaesthesia.

Author Contributions: EMH and EGR wrote the statistical code and performed the Bayesian analysis, interpreted the findings and wrote the manuscript; SG contributed to the writing of the manuscript; RP was the CI for the OPTIMISE study and contributed to the writing of the manuscript.

Patient consent: Written informed consent was obtained from all patients prior to surgery in the OPTIMISE trial.

Ethical approval: OPTIMISE was approved by the East London and City Research Ethics Committee and the Medical and Healthcare Products Regulatory Agency.

Data sharing agreement: Statistical code is available in the Supplementary Material.

For peer review only

Introduction

The traditional statistical approach of null hypothesis testing dominates clinical trial design and analysis. In this *frequentist* framework, conclusions are drawn using *p*-values and confidence intervals which have been generated to test a specific hypothesis (usually a null hypothesis of exactly zero treatment difference). It requires that a correct conclusion be drawn with a high level of probability (statistical power) from a notional set of repetitions of the trial.

The problems with this approach are well described (e.g., [1-4]). Frequentist results (p-values and confidence intervals) do not have simple or intuitive interpretations, and have often been misused and misinterpreted in clinical trials (see [2]). Randomised controlled trial results are often divided into "significant" and "non-significant", usually based on a p-value < 0.05. A statistically non-significant result for the primary endpoint is generally interpreted as the new treatment being "unsuccessful" (see [5, 6]). This is often an incorrect interpretation, and was highlighted by Altman and Bland [7], "absence of evidence is not evidence of absence". Randomised controlled trials are regarded as the gold standard of evidence and their results often inform clinical guidelines and practice. The misuse and misinterpretation of *p*-values (and confidence intervals) may result in the abandonment of potentially beneficial treatments. McShane et al. [4] recommended that null hypothesis testing should be abandoned altogether, and one peer-reviewed journal has banned its use [8].

Bayesian statistical methods provide an alternative framework for statistical modelling, and are increasingly being used in randomised controlled trials (e.g., [9-11]). The Bayesian approach can be used at the design stage and for data analysis. Bayesian statistics provide a formal method for combining pre-existing information with data that is collected in the clinical trial into the analysis so that the current state of knowledge can be updated. This is particularly useful for clinical research given that advances in health care usually occur through incremental gains in knowledge. The Bayesian approach may also provide results that are easier to interpret than the frequentist approach since it computes the probability of various values of the treatment effect, given the data.

Pre-existing information is incorporated into the analysis via a *prior distribution*. This is a probability distribution that accounts for uncertainty in an unknown parameter, for example, the treatment effect. The frequentist approach assumes the parameter to have a fixed value. Some are put off by this aspect of the Bayesian approach, expressing a concern that it is not scientifically objective. However, any statistical method, including traditional frequentist methods, involve subjective choices. For instance, the arbitrary set 5% significance level in the frequentist approach. Moreover, so-called *non-informative priors* can be used when there is little reliable previous information, or when one would prefer to mimic a frequentist analysis.

When clinical trial data are observed, the prior distribution is updated to become the *posterior distribution*, which summarises the current knowledge. The posterior distribution of the treatment effect provides the relative credibility of the range of treatment effect values, given the trial data. Calculation of the posterior distribution can often be straightforward, however, many practical examples require computationally intensive approaches. Part of the reason Bayesian statistics is becoming more popular is due to the advances in computing power and computational techniques. The posterior distribution can be used to answer questions of direct relevance to decision-making, such as "What is the probability that the new treatment is more successful than the current treatment?" Frequentist methods are limited as to what research questions they can answer, and cannot directly provide answers to clinically important questions.

For peer review only

Rather than using confidence intervals (CIs), Bayesian statistics can use highest posterior density intervals (HDI), which provide a range of values for the treatment effect for a certain level of posterior probability. Although frequentist CI and Bayesian HDI may sometimes be numerically similar, their interpretation is different. A 95% HDI says, given the observed data, there is a 95% probability that the true parameter value falls within this interval. The frequentist interpretation of a 95% CI says, if this trial were to be repeated many times and CIs were calculated, 95% of these intervals would contain the true parameter value.

In this article we provide an example of how a simple Bayesian analysis of a binary primary outcome could have been used instead of (or in addition to) traditional frequentist methods, to give a better insight into clinical application of the results. We analyse a randomised controlled trial of two treatments for patients undergoing major gastrointestinal surgery, to arrive at a conclusion that may be more clinically interpretable and useful, and also provide a more positive message from the trial.

Methods

Data and Study Design

The Optimisation of Cardiovascular Management to Improve Surgical Outcome (OPTIMISE) trial [12] was a pragmatic, observer-blinded, multi-centre, randomised controlled trial of 734 high-risk patients (aged 50 years and older) that had undergone major gastrointestinal surgery in 17 acute care hospitals in the United Kingdom. Previous research suggested that postoperative outcomes may be improved by the use of cardiac output monitoring to guide the administration of intravenous fluid and inotropic drugs in the time around surgery [13]. Patients were randomly assigned (1:1) to a cardiac output-guided hemodynamic therapy algorithm for intravenous fluid and inotropic drug administration during and in the 6 hours following surgery (intervention; n=368) or to usual care (n=366). The primary outcome was a composite of predefined 30-day moderate or major complications and mortality.

The primary outcome occurred in 158/364 (43.4%) usual care and 134/366 (36.6%) intervention patients. Using the traditional frequentist approach, the relative risk (RR) was found to be 0.84 (95% CI: 0.70, 1.01) and absolute risk reduction was 6.8% (95% CI: −0.3%, 13.9%), with p=0.07. This was interpreted as there being insufficient evidence that the RR for complication/death at 30 days is different to 1. Thus, the authors concluded, “use of a cardiac output–guided hemodynamic therapy algorithm compared with usual care did not reduce a composite outcome of complications and 30-day mortality.” [12]

Patient involvement

Patients were not involved in the re-analysis of the OPTIMISE trial.

Statistical Methods

By repeating the same analysis using Bayesian methods we can find an alternative way to model and interpret these data. The Bayesian analysis involves specifying a prior distribution for the primary outcome rate in each treatment arm, and using the OPTIMISE trial data to update those prior distributions to become posterior distributions. From the posterior distributions we calculate the relative risk (RR) or absolute risk difference and 95% HDI. We also use the posterior distribution to calculate the probability that the intervention is superior (RR < 1 or absolute risk difference < 0).

We can extend this idea by specifying a clinically significant effect size. For instance, a group of clinicians or patients could decide that a RR must differ from the null by, say, 10% to be clinically significant. This "region of practical equivalence" (ROPE) can be used to make probability statements about the likely clinical significance of effect sizes. For illustrative purposes we specify the ROPEs to be 0.9 to 1.1 for the RR and -0.05 to 0.05 (-5% to 5%) for the absolute risk difference. These ROPEs may not be reasonable in practice, and should be developed based on clinician, patient and health economists' feedback.

Additionally, we can simulate the outcomes of future patients using the updated estimates of the probabilities of having the primary outcome (from the posterior distribution) to predict the proportion of cases in which using the intervention would result in no complication/death whilst not using the intervention would.

We used two different prior distributions in separate analyses to check the robustness of our conclusions to our prior distribution assumptions. We used Beta distributions for the priors as these allowed the posterior distribution to be calculated more easily for our data. Initially we used an uninformative prior for both arms, the Beta(1,1) distribution, which assumes that the proportions for the composite outcome are uniform in each group (see Figure 1, top panel). Whilst some values of the composite outcome rate are more likely than others, an uninformative prior may be used to approximate a frequentist analysis. An evidence-based prior was specified using results from a pre-existing meta-analysis ([12, 13]) which had information on both treatment arms. The evidence-based prior distributions for the control and intervention arm are shown in Figure 1. Further details on how these priors were derived are displayed in the Supplementary Material.

The Bayesian analysis was conducted in R version 3.4.1 and additional information on the algorithm settings and the code for the Bayesian analysis is available in the Supplementary Material. Readers can perform the analysis here: https://argoshare.is.ed.ac.uk/bayesian_two_proportions/

[Figure 1 here]

Results

[Figure 2 here]

The results differ slightly between the uninformative and evidence-based priors, particularly for the probability of lying in the ROPE. Under the uninformative prior, the posterior mean RR is 0.85 (95% HDI: 0.70, 1), which is approximately the same as the frequentist analysis. Using the evidence-based

priors, the posterior mean RR is 0.81 (95% HDI: 0.67, 0.94). The probability that the intervention group has a lower incidence of the composite endpoint is 97% and 99.5%, assuming an uninformative and evidence-based prior, respectively (Figure 2). The posterior probability of different effect sizes (RRs) occurring is shown in Figure 3 (for both priors).

Based on the RR ROPE, the probability of the two arms being clinically equivalent was 24% and 9% under the uninformative and evidence-based priors, respectively. Alternatively, the probability of there being a clinically relevant difference in the RR of the composite endpoint between the two groups was 76% and 91% for the uninformative and evidence-based priors, respectively.

Using an uninformative prior, the posterior mean of the absolute risk difference/reduction was 6.76% (95% HDI: 0%, 13.8%), which is approximately the same as the frequentist analysis. Using the evidence-based prior, this value was 8.55% (95% HDI: 2.1%, 15.1%). When the ROPE was defined as an absolute risk difference of -5% to 5%, the probabilities of the trial arms being equivalent were 31% and 14% (69% and 86% probability of there being a clinically relevant difference) under the uninformative and evidence-based priors, respectively.

Using a posterior prediction from the Bayesian model, the probability that a patient in the intervention group did not have a complication/death when a patient in the control group did have a complication/death was 27.4% (uninformative prior) and 28.4% (evidence-based prior).

[Figure 3 here]

Discussion

In this work we have performed a Bayesian re-analysis of the OPTIMISE trial [12] to demonstrate how a Bayesian approach can more easily answer clinical questions of interest and inform decision making. Our conclusions from the analysis are given in terms of probabilities that a benefit exists. The principal finding of this analysis was that the use of a Bayesian analytical approach provided a different interpretation of the findings of the OPTIMISE trial, suggesting patient benefit from the intervention.

The frequentist analysis conducted by Pearse et al. [12] identified no significant difference for 30-day complications/death between the treatment arms and deemed the trial to be underpowered. The Bayesian posterior mean estimates and 95% HDI gave similar results to the frequentist analysis as expected, with more optimistic results produced by the evidence-based prior. (Previous evidence had suggested that the intervention was superior.) The primary advantage here of using the Bayesian approach is that we can explicitly obtain the posterior distribution of the primary outcome rates for each arm, and from this the treatment effect distribution, which can be interpreted directly and intuitively. From these distributions we can calculate the probability that the new treatment is superior. A frequentist analysis, particularly of an underpowered trial, provides little help for clinicians to make the necessary decisions regarding future treatment and also runs the risk of concluding no difference between treatments (based on a non-significant p-value), even when there is in reality a useful difference.

For peer review only

For a reasonably large trial, different priors should have little effect on the posterior distributions as the data are contributing a large amount of information, and generally outweigh the contribution of the prior information. In this study, altering the priors had little impact on the results, most likely due to the fairly large sample size. The evidence-based prior, which used information from 21 small studies (sample size ranged from 34 to 390 patients) that were mostly in favour of the intervention and was quite precise, produced a slightly smaller RR and provided more certainty that the intervention was superior. Since these were small studies, one may wish to down weight the influence of these studies in the prior to consider the risk of bias. Plots of the priors overlaid with the posteriors for the evidence-based priors are provided in the Supplementary Material (Figure S3) and show that the prior for the intervention was too optimistic.

The Bayesian analyses that we have presented here are simple, and repeat the primary analysis of the OPTIMISE trial to demonstrate the usefulness of the Bayesian approach. A Bayesian analysis cannot salvage a badly designed or conducted trial. Instead, the Bayesian approach can be used to produce results that are more easily interpreted and avoids the dichotomisations that occur in significance testing.

Conclusion

Using the results from a standard statistical analysis, the OPTIMISE trial authors concluded that the use of the intervention compared with usual care did not reduce a composite outcome of complications and 30-day mortality [12]. Although the trial was not powered to detect a statistically significant difference between the treatment arms for the observed effect size, by using Bayesian analyses we were able to demonstrate that the intervention was superior to the control with 97-99.5% probability. These results are much more clinically useful than those that are given by the traditional approach.

References

[1] Simmons JP, Nelson LD, Simonsohn U. False-positive psychology: Undisclosed flexibility in data collection and analysis allows presenting anything as significant. Psychol Sci. 2011 Oct 17; 22(11):1359-1366.

[2] Greenland S, Senn SJ, Rothman KJ, Carlin JB, Poole CP, Goodman SN, Altman DG. Statistical tests, *P* values, confidence intervals, and power: a guide to misinterpretations. Eur J Epidemiol. 2016 May 21; 31: 337-350.

[3] Wasserstein RL, Lazar NA. The ASA's statement on p-values: context, process and purpose. The American Statistician. 2016 June 09; 70(2): 129-133

[4] McShane BB, Gal D, Gelman A, Robert C, Tackett JL. Abandon Statistical Significance. arXiv preprint arXiv:1709.07588. 2017 Sep 21.

For peer review only - http://bmjopen.bmj.com/site/about/guidelines.xhtml

[5] Alderson P, Chalmers I. Survey of claims of no effect in abstracts of Cochrane reviews. BMJ. 2003 Mar 01; 326(7387):475.

[6] Greenland S. Null misinterpretation in statistical testing and its impact on health risk assessment. Prev Med. 2011 Aug 17; 53:225–8.

[7] Altman DG, Bland JM. Statistics notes: Absence of evidence is not evidence of absence. BMJ. 1995 Aug 19; 311(7003):485.

[8] Trafimow D, Marks M. Editorial. Basic Appl. Soc. Psych. 2015 Feb 12; 37(1): 1-2

[9] Morris RK, Malin GL, Quinlan-Jones E, Middleton LJ, Hemming K, Burke D, et al. Percutaneous vesicoamniotic shunting versus conservative management for fetal lower urinary tract obstruction (PLUTO): a randomised trial. Lancet. 2013 Aug 13; 382(9903): 1496-1506.

[10] Laptook AR, Shankaran S, Tyson JE, Munoz B, Bell EF, Goldberg RN, et al. Effect of Therapeutic Hypothermia Initiated After 6 Hours of Age on Death or Disability Among Newborns With Hypoxic-Ischemic Encephalopathy A Randomized Clinical Trial. JAMA. 2017 October 31; 318(16): 1550-1560.

[11] Reardon MJ, Van Miegham NM, Popma JJ, Kleiman NS, Sondergaard L, Mumtaz M, et al. Surgical or Transcatheter Aortic-Valve Replacement in Intermediate-Risk Patients. NEJM. 2017 Mar 17; 376(14): 1321-1331.

[12] Pearse RM, Harrison DA, MacDonald N, Gillies MA, Blunt M, Ackland G, et al. Effect of a Perioperative, Cardiac Output–Guided Hemodynamic Therapy Algorithm on Outcomes Following Major Gastrointestinal Surgery A Randomized Clinical Trial and Systematic Review. JAMA. 2014 May 19; 311(21): 2181-2190.

[13] Grocott MP, Dushianthan A, Hamilton MA, Mythen MG, Harrison D, Rowan K; Optimisation Systematic Review Steering Group. Perioperative increase in global blood flow to explicit defined goals and outcomes following surgery. *Cochrane Database Syst Rev*. 2012 Nov 14;11:CD004082.

For peer review only

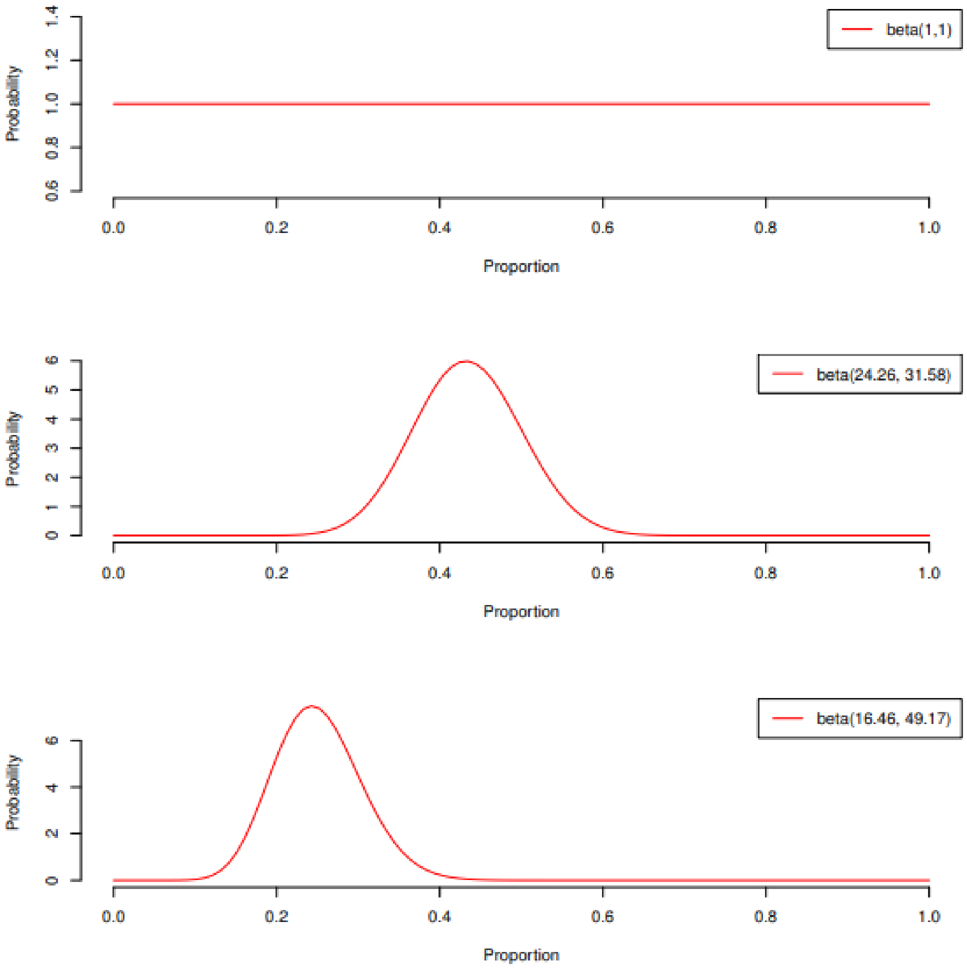

Figure 1. Prior distributions used in Bayesian reanalysis of OPTIMISE. The top panel is an uninformative prior distribution, beta(1,1), that was used for both treatment arms. The middle and bottom panels show the evidence-based priors that were derived using a meta-analysis. The middle panel is the prior for the control arm (beta(24.26, 31.58)) and the bottom panel is the prior for the intervention arm (beta(16.46, 49.17)).

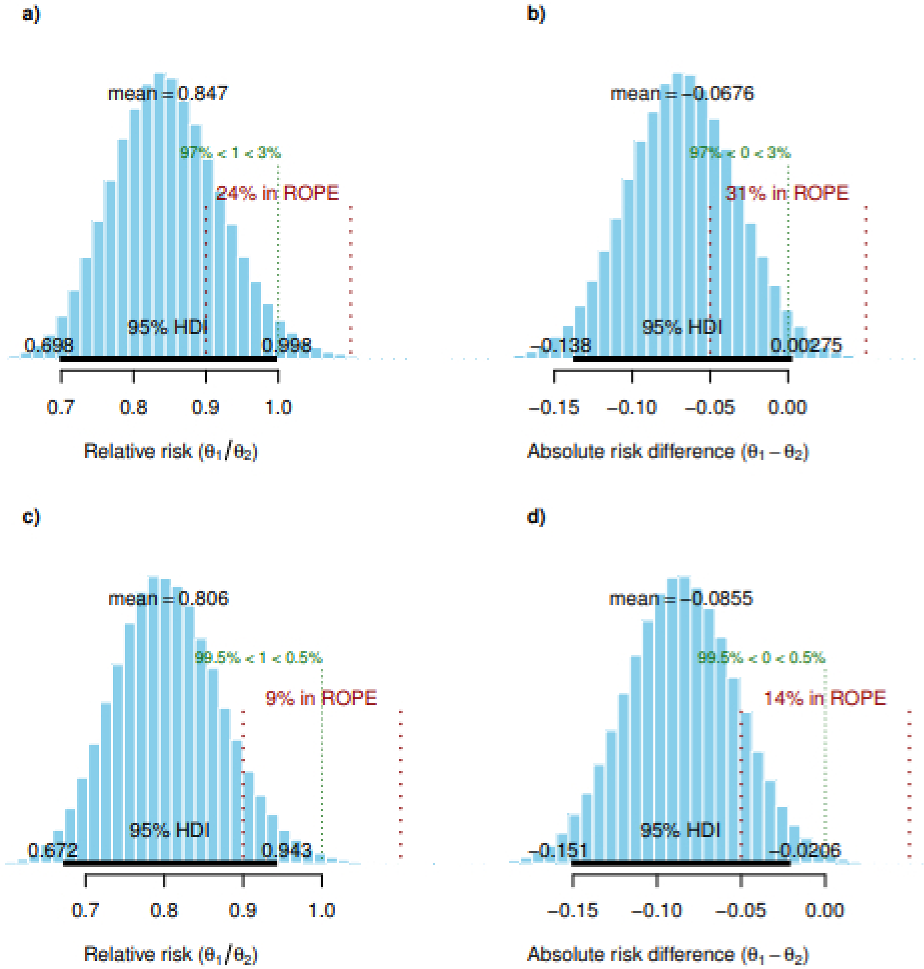

Figure 2. Posterior distributions of relative risk (RR) and absolute risk difference for the uninformative prior and evidence-based priors. θ1 and θ2 are the composite outcome rates in the intervention and control arms, respectively. a) Posterior distribution of the RR of the primary outcome using an uninformative prior; b) posterior distribution of the absolute risk difference in the primary outcome using an uninformative prior; c) posterior distribution of the RR of the primary outcome using the evidence-based priors; d) posterior distribution of the absolute risk difference in the primary outcome using the evidence-based priors. A RR>1 indicates that the intervention is more harmful. A positive value of the absolute risk difference indicates that the intervention is more harmful. The 95% highest posterior density interval (HDI) is shown as the thick black horizontal line with the boundaries written above the line. The region of practical equivalence (ROPE) is shown in red (with dotted vertical lines). The posterior probability of RR<1 and RR>1, and an absolute difference <0 and >0 is shown in green above the ROPE.

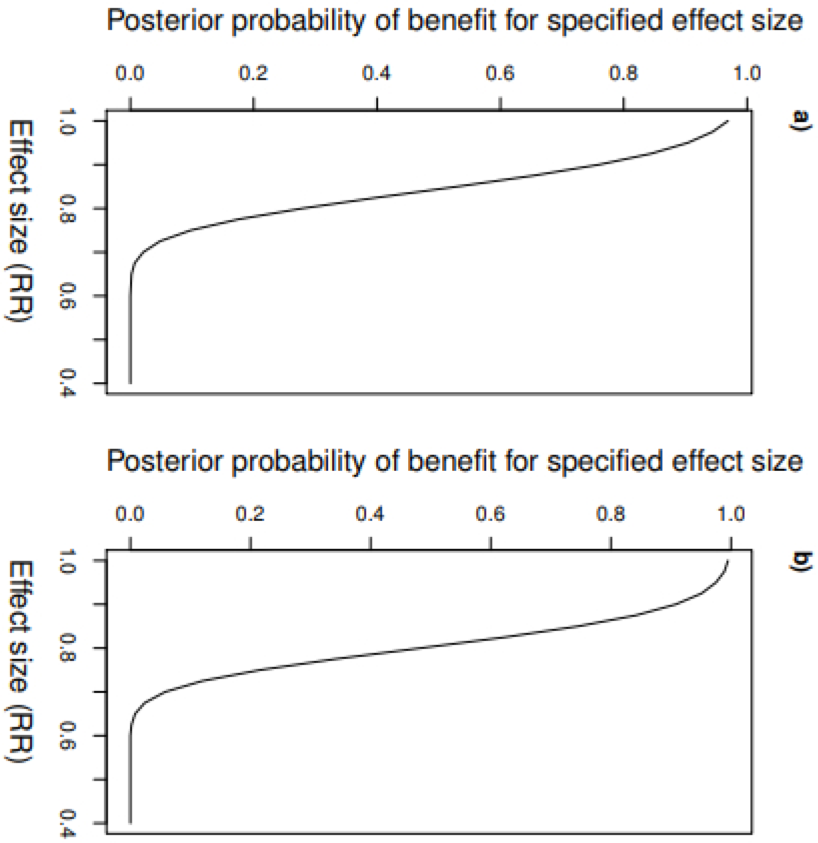

Figure 3. Posterior probability of specified effect sizes, i.e. Probability RR≤ effect size, using (a) uninformative prior, (b) evidence-based priors. A RR<1 indicates that the primary outcome rate is smaller in the intervention arm compared to the control arm.

Supplementary Material

Algorithms settings and code for Bayesian analysis

The Bayesian analysis was conducted in R version 3.4.1 using the *rjags* package [1], which provides an interface to the JAGS library (JAGS version 4.3.0) to perform Bayesian data analysis. JAGS (Just Another Gibbs Sampler) was derived from BUGS (Bayesian analysis Using Gibbs Sampling; see [2] for details on the BUGS project) and uses Markov Chain Monte Carlo (MCMC) to generate samples from the posterior distribution so that inference on the parameters of interest may be obtained. The posterior distribution was obtained using a sample of 200 000 estimates of the primary outcome probability for each arm (used 3 chains each of length 66667, with a burn-in period of 1000 iterations and 500 iterations were used to adapt/tune the samplers). Model convergence was checked using trace plots, density plots, and the Gelman-Rubin diagnostic [3]. The MCMC code is presented below in "R code 1".

Since we used Beta distributions for the priors, and have binary data that is modelled by a binomial distribution, we are able to obtain an algebraic expression to calculate the posterior distribution. The Beta distribution prior is a *conjugate prior* for the binomial distribution, which means that the posterior distribution is in the same probability distribution family as the prior distribution (Beta distribution). We can update the Beta distribution parameters using the data from the trial:

$$Beta(\alpha_t + y_t, \beta_t + N_t - y_t)$$

where $\mathrm{Beta}(\alpha_{t,}, \beta_t)$ is the prior for treatment arm *t;* y_t are the number of complications/deaths at 30 days for arm *t*; and N_t are the number of patients randomised to arm *t*. We can simulate samples from this distribution for each arm (simulating from the posterior) and examine the relative risks (RR) and absolute risk differences. This code is presented in "R code 2".

w only

R code 1. Code for posterior simulations using an MCMC algorithm

```
# OPTIMISE trial in a Bayesian framework
# JAMA. 2014;311(21):2181-2190. doi:10.1001/jama.2014.5305


remove(list=ls())


#specify your working directory
setwd("H:/Documents/optimise ii")



# Primary outcome: composite of 30-day moderate or major complications and mortality
N1 <- 366
y1 <- 134
```

```
N2 <- 364

y2 <- 158

# N1 is total number in the Cardiac Output–Guided Hemodynamic Therapy Algorithm (intervention) group

# y1 is number with the outcome in the Cardiac Output–Guided Hemodynamic Therapy Algorithm (intervention) group

# N2 is total number in usual care (control) group

# y2 is number with the outcome in usual care (control) group


# Risk ratio

(y1/N1)/(y2/N2)


#frequentist analysis

library(epitools)

riskratio(c(N1-y1, y1, N2-y2, y2), rev="rows", method="boot", replicates=100000)


# Using standard frequentist approach

# Risk ratio (bootstrapped 95% confidence intervals) = 0.84 (0.70-1.01)

# p=0.07 (Fisher exact p-value)



#-----------------------------------------------------------------------------


# Bayesian analysis of same ratio

# Base script from Kruschke, J. K. (2011). Doing Bayesian Data Analysis, Academic Press / Elsevier.


#load required packages (install first if necessary)

require(rjags)

library(BEST)




# The model will be specified with uninformative prior distributions (beta(1,1,).


# Plot prior beta(1,1)

par(mfrow=c(1,1))

plot(seq(0, 1, length.out=100), dbeta(seq(0, 1, length.out=100), 1,1),

                    type="l", xlab="Proportion",

                    ylab="Probability",
```

For peer review only

```
main="OPTIMISE Composite Primary Outcome Prior distribution",
frame=FALSE, col="red", oma=c(6,6,6,6))
legend("topright", legend="beta(1,1)", lty=1, col="red", inset=0)

#------------------------------------------------------------------------------

# Specify the MODEL.
modelString = "
# JAGS model specification begins here...
model {
# Likelihood. Each complication/death is Bernoulli.
for ( i in 1 : N1 ) { y1[i] ~ dbern( theta1 ) }
for ( i in 1 : N2 ) { y2[i] ~ dbern( theta2 ) }
# Prior. Independent beta distributions. Change this for informative prior
theta1 ~ dbeta( 1,1) #intervention
theta2 ~ dbeta( 1,1) #control
}
# ... end JAGS model specification
" # close quote for modelstring

# Write the modelString to a file, using R commands:
writeLines(modelString,con="H:/Documents/optimise ii/model.txt")


#------------------------------------------------------------------------------
# THE DATA.

# Specify the data in a form that is compatible with JAGS model, as a list:
dataList =      list(
      N1 = N1 ,
      y1 = c(rep(1, y1), rep(0, N1-y1)),
      N2 = N2 ,
      y2 = c(rep(1, y2), rep(0, N2-y2))
)

#------------------------------------------------------------------------------
```

For peer review only

```
# INTIALIZE THE CHAIN.


# Can be done automatically in jags.model() by commenting out inits argument.

# Otherwise could be established as:

# initsList = list( theta1 = sum(dataList$y1)/length(dataList$y1) ,

#                   theta2 = sum(dataList$y2)/length(dataList$y2) )


#------------------------------------------------------------------------------

# RUN THE CHAINS.


#specify the MCMC parameters

parameters = c( "theta1" , "theta2" )     # The parameter(s) to be monitored.

adaptSteps = 500              # Number of steps to "tune" the samplers.

burnInSteps = 1000            # Number of steps to "burn-in" the samplers.

nChains = 3                   # Number of chains to run.

numSavedSteps=200000          # Total number of steps in chains to save.

thinSteps=1                   # Number of steps to "thin" (1=keep every step).

nIter = ceiling( ( numSavedSteps * thinSteps ) / nChains ) # Steps per chain.




# Create, initialize, and adapt the model:

jagsModel = jags.model( "H:/Documents/optimise ii/model.txt" , data=dataList , # inits=initsList ,

                        n.chains=nChains , n.adapt=adaptSteps )

# Burn-in:

cat( "Burning in the MCMC chain...n" )


#run the MCMC

update( jagsModel , n.iter=burnInSteps )


# The saved MCMC chain:

cat( "Sampling final MCMC chain...n" )

codaSamples = coda.samples( jagsModel , variable.names=parameters ,

                        n.iter=nIter , thin=thinSteps )


# resulting codaSamples object has these indices:
```

For peer review only

```
#   codaSamples[[ chainIdx ]][ stepIdx , paramIdx ]



#check MCMC has converged

plot(codaSamples)

summary(window(codaSamples, start = burnInSteps))

# Gelman-Rubin diagostic - factor of 1 means that between chain variance and within chain variance are equal.
Values <=1.1 suggest convergence

gelman.diag(codaSamples)

#Check whether the low chain reduction is stable.

gelman.plot(codaSamples)

#Burn-in appear sufficient (choose the iteration after which the lines stay below 1.1)



#------------------------------------------------------------------------------

# EXAMINE THE RESULTS.


# Convert coda-object codaSamples to matrix object for easier handling.

# This concatenates the different chains into one long chain.

# Result is mcmcChain[ stepIdx , paramIdx ]

mcmcChain = as.matrix( codaSamples )



theta1Sample = mcmcChain[,"theta1"] # Put sampled values in a vector - INTERVENTION ARM.

theta2Sample = mcmcChain[,"theta2"] # Put sampled values in a vector - CONTROL ARM




# Plot a histogram of the posterior differences of theta values.

thetaRR = theta1Sample / theta2Sample # Relative risk

thetaDiff = theta1Sample - theta2Sample # Absolute risk difference


# 2x1 plots

par(mfrow=c(2,1))


# plot posterior of RR

par(mar=c(4, 4.1, 4.1, 4))
```

For peer review only

```
plotPost( thetaRR , xlab= expression(paste("Relative risk (", theta[1]/theta[2], ")")) ,
        compVal=1.0, ROPE=c(0.9, 1.1),
        main="OPTIMISE Composite Primary Outcome
Posterior distribution of relative risk", cex.main=1, cex=1, cex.lab=1, xlab=c(0.6,1.4))

#Plot absolute risk difference
plotPost( thetaDiff , xlab=expression(paste("Absolute risk difference (", theta[1]-theta[2], ")")) ,
        compVal=0.0, ROPE=c(-0.05, 0.05),
        main="OPTIMISE Composite Primary Outcome
Posterior distribution of absolute risk difference", cex.main=1, cex=1, cex.lab=1, xlab=c(-0.25, 0.15))



#------------------------------------------------------------------------------

# Use posterior prediction to determine proportion of cases in which
# using the intervention would result in no complication/death
# while not using the intervention would result in complication death

chainLength = length( theta1Sample )

# Create matrix to hold results of simulated patients:
yPred = matrix( NA , nrow=2 , ncol=chainLength )

# For each step in chain, use posterior prediction to determine outcome
for ( stepIdx in 1:chainLength ) { # step through the chain
        # Probability for complication/death for each "patient" in intervention group:
        pDeath1 = theta1Sample[stepIdx]
        # Simulated outcome for each intervention "patient"
        yPred[1,stepIdx] = sample( x=c(0,1), prob=c(1-pDeath1,pDeath1), size=1 )
        # Probability for complication/death for each "patient" in control group:
        pDeath2 = theta2Sample[stepIdx]
        # Simulated outcome for each control "patient"
        yPred[2,stepIdx] = sample( x=c(0,1), prob=c(1-pDeath2,pDeath2), size=1 )
}
```

```
# Now determine the proportion of times that the intervention group has no complication/death
# (y1 == 0) and the control group does have a complication or death (y2 == 1))
(pY1eq0andY2eq1 = sum( yPred[1,]==0 & yPred[2,]==1 ) / chainLength)

#proportion of times intervention has complication/death where control group does not
(pY1eq1andY2eq0 = sum( yPred[1,]==1 & yPred[2,]==0 ) / chainLength)

#proportion of times where neither arm has complication/death
(pY1eq0andY2eq0 = sum( yPred[1,]==0 & yPred[2,]==0 ) / chainLength)

#proportion of times where both arms have complication/death
(pY10eq1andY2eq1 = sum( yPred[1,]==1 & yPred[2,]==1 ) / chainLength)
```

For peer review only

R Code 2. Code for exact posterior calculation

```
remove(list=ls())

###uninformative prior

#prior for control group is beta(a1, b1)
a1=1
b1=1

#prior for treatment group is beta(a2, b2)
a2=1
b2=1

#control event/total is z1/N1
z1=158
N1=364

#treatment event/total is z2/N2.
z2=134
N2=366

#simulate from the posterior for control group. posterior can be calculated exactly as beta(a1+z1, b1+N1-z1)
pc = rbeta(200000, a1+z1, b1+N1-z1)
#simulate from the posterior for intervention group. posterior can be calculated exactly as beta(a2+z2, b2+N2-z2)
pt = rbeta(200000, a2+z2, b2+N2-z2)


thetaRR = pt / pc # Relative risk
thetaDiff = pt - pc # Absolute risk difference

#########################################################################
#########################################################################
#evidence-based prior

#prior for control group is beta(a1, b1)
a1=24.26
b1=31.58


#prior for treatment group is beta(a2, b2)
a2=16.46
b2=49.17

#control event/total is z1/N1
z1=158
N1=364

#treatment event/total is z2/N2.
z2=134
N2=366

#simulate from the posterior for control group. posterior can be calculated exactly as beta(a1+z1, b1+N1-z1)
```

For peer review only

```
pcc = rbeta(200000, a1+z1, b1+N1-z1)
#simulate from the posterior for intervention group. posterior can be calculated exactly as beta(a2+z2, b2+N2-z2)
ptt = rbeta(200000, a2+z2, b2+N2-z2)



thetaRR_ev = ptt / pcc # Relative risk
thetaDiff_ev = ptt - pcc # Absolute risk difference


#######################################################################

#plot posterior distributions

require(rjags)
library(BEST)

# 2x2 plots
par(mfrow=c(2,2))

# plot posterior for uninformative prior, RR
par(mar=c(4, 4.1, 4.1, 4))
plotPost( thetaRR , xlab= expression(paste("        Relative risk (", theta[1]/theta[2], ")")) ,
        compVal=1.0, ROPE=c(0.9, 1.1),
        main="a)", adj=0,cex.main=1, cex=1, cex.lab=1, xlab=c(0.6,1.4))

# plot posterior for uninformative prior, absolute risk difference
plotPost( thetaDiff , xlab=expression(paste("Absolute risk difference (", theta[1]-theta[2], ")")) ,
        compVal=0.0, ROPE=c(-0.05, 0.05),
        main="b)", adj=0,cex.main=1, cex=1, cex.lab=1, xlab=c(-0.25, 0.15))

# plot posterior for evidence-based prior, RR
par(mar=c(4, 4.1, 4.1, 4))
plotPost( thetaRR_ev , xlab= expression(paste("        Relative risk (", theta[1]/theta[2], ")")) ,
        compVal=1.0, ROPE=c(0.9, 1.1),
        main="c)", adj=0,cex.main=1, cex=1, cex.lab=1, xlab=c(0.6,1.4))

# plot posterior for evidence-based prior, absolute risk difference
plotPost( thetaDiff_ev , xlab=expression(paste("Absolute risk difference (", theta[1]-theta[2], ")")) ,
        compVal=0.0, ROPE=c(-0.05, 0.05),
        main="d)", adj=0,cex.main=1, cex=1, cex.lab=1, xlab=c(-0.25, 0.15))
```

For peer review only

Evidence-based Prior

An evidence-based prior was specified using results from a pre-existing meta-analysis ([4,5] – not including the OPTIMISE results) which had information on both treatment arms. The R Bayesian evidence synthesis Tools (RBesT; [6]) were used to perform a Bayesian meta-analysis, which accounts for the uncertainty of the population mean and between-trial heterogeneity (using the *gMAP* function in R (version 3.4.1)). The meta-analysis of the control and intervention arms are displayed in Figures S1 and S2, respectively.

For the control arm, the meta-analysis gave a mean estimate of 0.432 (95% HDI: 0.306, 0.563). Using the *betaExpert* function (from the *prevalence* package [7]) in R version 3.4.1 (setting the mean from the meta-analysis to the mode of the beta distribution, and using the 95% HDI) gave a beta(24.26,31.58) distribution. This was used as the prior for the control arm.

For the intervention arm, the meta-analysis gave a mean estimate of 0.243 (95% HDI: 0.148, 0.358). Using the *betaExpert* function [7] in R (setting the mean from the meta-analysis to the mode of the beta distribution, and using the 95% HDI) gave a beta(16.46, 49.17) distribution. This was used as the prior for the intervention arm.

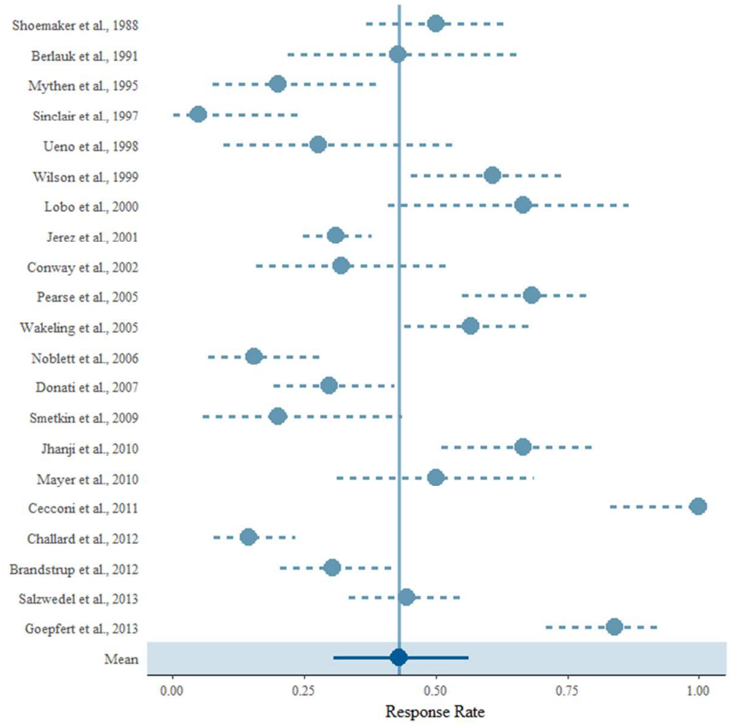

Figure S1.Forest plot of control arm using meta-analysis from Pearse et al. [5]. Dashed lines represent the stratified estimates with 95% HDI. Solid line represents the mean estimate (with 95% HDI))

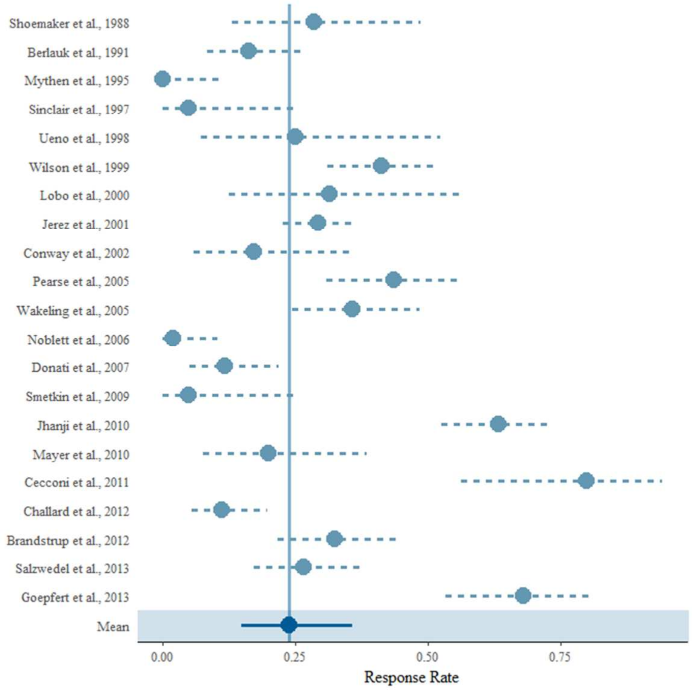

Figure S2. Forest plot of intervention arm using meta-analysis from Pearse et al. [5]. Dashed lines represent the stratified estimates with 95% HDI. Solid line represents the mean estimate (with 95% HDI))

For peer review only

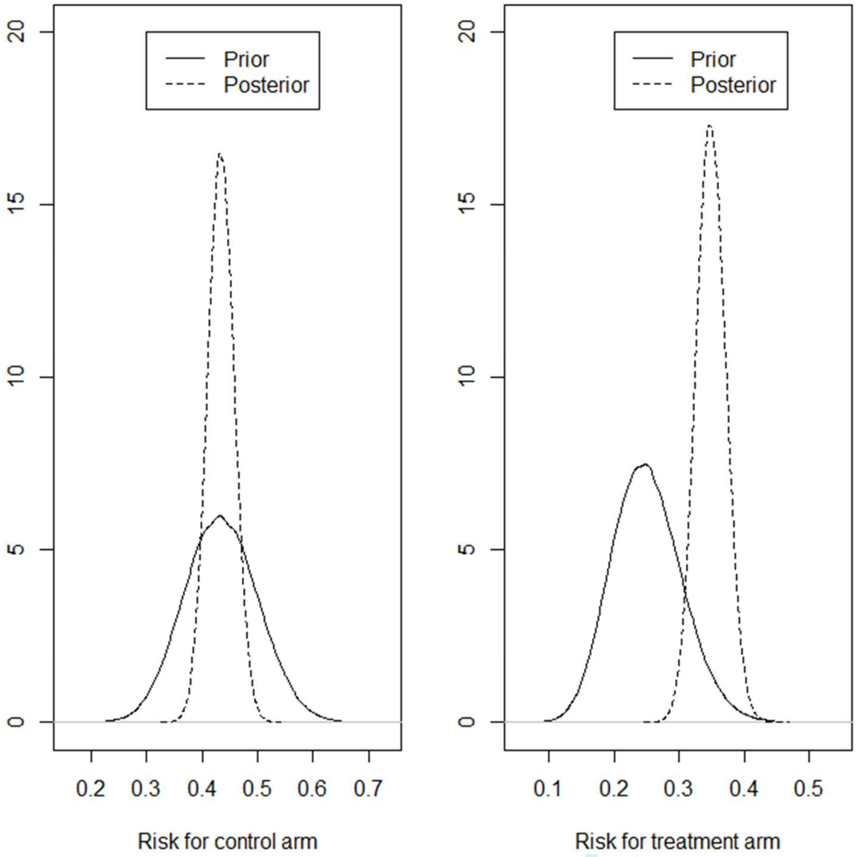

Figure S3. Plot of the evidence-based priors overlaid with the posteriors for each treatment arm

References

[1] Plummer M. JAGS: A program for analysis of Bayesian graphical models using Gibbs sampling. Proceedings of the 3rd International Workshop on Distributed Statistical Computing (DSC 2003). March 2003. p. 20–2.

[2] Lunn D, Spiegelhalter D, Thomas A, Best N. The BUGS project: Evolution, critique and future directions. Statistics in Medicine. 2009 October 13; 28(25): 3049-3067

[3] Gelman A, Rubin DB. Inference from Iterative Simulation Using Multiple Sequences. Statistical Science. 1992; 7(4): 457-472.

[4] Grocott MP, Dushianthan A, Hamilton MA, Mythen MG, Harrison D, Rowan K; Optimisation Systematic Review Steering Group. Perioperative increase in global blood flow to explicit defined goals and outcomes following surgery. *Cochrane Database Syst Rev*. 2012 Nov 14;11:CD004082.

[5] Pearse RM, Harrison DA, MacDonald N, Gillies MA, Blunt M, Ackland G, et al. Effect of a Perioperative, Cardiac Output–Guided Hemodynamic Therapy Algorithm on Outcomes

For peer review only - http://bmjopen.bmj.com/site/about/guidelines.xhtml

Following Major Gastrointestinal Surgery A Randomized Clinical Trial and Systematic Review. JAMA. 2014 May 19; 311(21): 2181-2190.

[6] Weber S. (2017). RBesT: R Bayesian Evidence Synthesis Tools. R package version 1.2-3. https://CRAN.R-project.org/package=RBesT

[7] Devleesschauwer B, Torgerson P, Charlier J, Levecke B, Praet N, Roelandt S, et al. (2014). prevalence: Tools for prevalence assessment studies. R package version 0.4.0. http://cran.r-project.org/package=prevalence

For peer review only

BMJ Open

Perioperative haemodynamic therapy for major gastrointestinal surgery: The effect of a Bayesian approach to interpreting the findings of randomised controlled trials

Journal:	*BMJ Open*
Manuscript ID	bmjopen-2018-024256.R1
Article Type:	Research
Date Submitted by the Author:	02-Oct-2018
Complete List of Authors:	Ryan, Elizabeth; University of Warwick, Warwick Clinical Trials Unit; University of Birmingham, Cancer Research UK Clinical Trials Unit<br>Harrison, Ewen; Univeristy of Edinburgh, Clinical Surgery<br>Pearse, Rupert; Queen Mary University of London, Barts and the London School of Medicine & Dentistry<br>Gates, Simon; University of Birmingham, Cancer Research UK Clinical Trials Unit; University of Warwick, Warwick Clinical Trials Unit
**Primary Subject Heading**:	Research methods
Secondary Subject Heading:	Gastroenterology and hepatology, Surgery
Keywords:	Bayesian analysis, randomised controlled trial, SURGERY, peri-operative care

SCHOLARONE™
Manuscripts

Perioperative haemodynamic therapy for major gastrointestinal surgery: The effect of a Bayesian approach to interpreting the findings of randomised controlled trials

Elizabeth G. Ryan*†, Ewen M. Harrison*, Rupert M. Pearse, and Simon Gates

***joint first authors**

†Corresponding author
Dr Elizabeth G. Ryan
Cancer Research Clinical Trials Unit, Institute of Cancer and Genomic Sciences, University of Birmingham, Birmingham, United Kingdom B15 2TT
phone: (+44) 0121 414 3289
Email: mailto:E.G.Ryan@bham.ac.uk

Email addresses:
EGR: E.G.Ryan@bham.ac.uk
EMH: mail@ewenharrison.com
RMP: r.pearse@qmul.ac.uk
SG: S.Gates@bham.ac.uk

For peer review only

Abstract

Objective: The traditional approach of null hypothesis testing dominates the design and analysis of randomised controlled trials. This study aimed to demonstrate how a simple Bayesian analysis could have been used to analyse the OPTIMISE trial to obtain more clinically interpretable results.

Design, setting, participants, and interventions: The OPTIMISE trial was a pragmatic, multicentre, observed-blinded, randomised controlled trial of 734 high-risk patients undergoing major gastrointestinal surgery in 17 acute care hospitals in the United Kingdom. Patients were randomly allocated to a cardiac output-guided hemodynamic therapy algorithm for intravenous fluid and inotropic drug administration during and in the 6 hours following surgery (n=368) or to standard care (n=366). The primary outcome was a binary outcome consisting of a composite of predefined 30-day moderate or major complications and mortality.

Methods: We repeated the primary outcome analysis of the OPTIMISE trial using Bayesian statistical methods to calculate the probability that the intervention was superior, and the probability that a clinically relevant difference existed. We explored the impact of a flat prior and an evidence-based prior on our analyses.

Results: Although OPTIMISE was not powered to detect a statistically significant difference between the treatment arms for the observed effect size (RR=0.84, 95% CI: 0.70, 1.01; p=0.07), by using Bayesian analyses we were able to demonstrate that there was a 96.9% (flat prior) to 99.5% (evidence-based prior) probability that the intervention was superior to the control.

Conclusions: The use of a Bayesian analytical approach provided a different interpretation of the findings of the OPTIMISE trial (compared to the original frequentist analysis), and suggested patient benefit from the intervention. Incorporation of information from previous studies provided further evidence of a benefit from the intervention. Bayesian analyses can produce results that are more easily interpretable and relevant to clinicians and policy makers.

Trial registration: isrctn.org Identifier: ISRCTN04386758

Keywords: Bayesian analysis; randomised controlled trial; surgery; peri-operative care

Word count: 2894

Strengths and limitations

- The use of a Bayesian analytical approach provided a different interpretation of the findings of the OPTIMISE trial (compared to the original frequentist analysis).
- The Bayesian approach avoids the dichotomisation of results that occur in significance testing.
- Bayesian analyses can produce results that are more easily interpretable and relevant to clinicians and policy makers.
- The analysis only used simple Bayesian methods and more sophisticated approaches can be used, such as adjusting for covariates.

Funding: EGR and SG were supported by a Medical Research Council (MRC) Methodology Research Grant (Grant number: MR/N028287/1).

Competing Interests: RMP holds research grants, and has given lectures and/or performed consultancy work for Nestle Health Sciences, BBraun, Medtronic, Glaxo Smithkline, Intersurgical, and Edwards Lifesciences, and is a member of the Associate editorial board of the British Journal of Anaesthesia.

Author Contributions: EMH and EGR wrote the statistical code and performed the Bayesian analysis, interpreted the findings and wrote the manuscript; SG contributed to the writing of the manuscript; RP was the CI for the OPTIMISE study and contributed to the writing of the manuscript.

Patient consent: Written informed consent was obtained from all patients prior to surgery in the OPTIMISE trial.

Ethical approval: OPTIMISE was approved by the East London and City Research Ethics Committee and the Medical and Healthcare Products Regulatory Agency.

Data sharing agreement: Statistical code is available in the Supplementary Material.

For peer review only

Introduction

The traditional statistical approach of null hypothesis testing dominates clinical trial design and analysis. In this *frequentist* framework, conclusions are drawn using *p*-values and confidence intervals which have been generated to test a specific hypothesis (usually a null hypothesis of exactly zero treatment difference). It requires that a correct conclusion be drawn with a high level of probability (statistical power) from a notional set of repetitions of the trial.

The problems with this approach are well described (e.g., [1-4]). Frequentist results (*p*-values and confidence intervals) do not have simple or intuitive interpretations, and have often been misused and misinterpreted in clinical trials (see [2]). Randomised controlled trial results are often divided into "significant" and "non-significant", usually based on a *p*-value < 0.05. A statistically non-significant result for the primary endpoint is generally interpreted as the new treatment being "unsuccessful" (see [5, 6]). This is often an incorrect interpretation, and was highlighted by Altman and Bland [7], "absence of evidence is not evidence of absence". Randomised controlled trials are regarded as the gold standard of evidence and their results often inform clinical guidelines and practice. The misuse and misinterpretation of *p*-values (and confidence intervals) may result in the abandonment of potentially beneficial treatments. McShane et al. [4] recommended that null hypothesis testing should be abandoned altogether, and one peer-reviewed journal has banned its use [8].

Bayesian statistical methods provide an alternative framework for statistical modelling, and are increasingly being used in randomised controlled trials (e.g., [9-11]). The Bayesian approach can be used at the design stage and for data analysis. Bayesian statistics provide a formal method for combining pre-existing information with data that is collected in the clinical trial into the analysis so that the current state of knowledge can be updated. This is particularly useful for clinical research given that advances in health care usually occur through incremental gains in knowledge. The Bayesian approach may also provide results that are easier to interpret than the frequentist approach since it computes the probability of various values of the treatment effect, given the data.

Pre-existing information is incorporated into the analysis via a *prior distribution*. This is a probability distribution that accounts for uncertainty in an unknown parameter, for example, the treatment effect, before the data from the clinical trial has been incorporated. The frequentist approach assumes the parameter to have a fixed, but unknown value. Some are put off by this aspect of the Bayesian approach, expressing a concern that it is not scientifically objective. However, any statistical method, including traditional frequentist methods, involve subjective choices. For instance, the arbitrarily set 5% significance level in the frequentist approach. Moreover, so-called *non-informative priors* can be used when there is little reliable previous information, or when one would prefer to numerically mimic a frequentist analysis and avoid introducing external information into the analysis. These are often used as a default prior.

When clinical trial data are observed, the prior distribution is updated to become the *posterior distribution*, which summarises the current knowledge. The posterior distribution of the treatment effect provides the relative credibility of the range of treatment effect values, given the trial data. Calculation of the posterior distribution can often be straightforward, however, many practical examples require computationally intensive approaches. Part of the reason Bayesian statistics is becoming more popular is due to the advances in computing power and computational techniques. The posterior distribution can be used to answer questions of direct relevance to decision-making, such as "What is the probability that the new treatment is more successful than the current

For peer review only

treatment?" Frequentist methods are limited as to what research questions they can answer, and cannot directly provide answers to clinically important questions.

Rather than using confidence intervals (CIs), Bayesian statistics can use credible intervals, which provide a range of values for the treatment effect for a certain level of posterior probability. The highest posterior density interval (HDI) is the narrowest type of credible interval available for a specified probability. Although frequentist CI and Bayesian HDI may sometimes be numerically similar, their interpretation is different. A 95% HDI says, given the observed data, there is a 95% probability that the true parameter value falls within this interval. The frequentist interpretation of a 95% CI says, if this trial were to be repeated many times and CIs were calculated, 95% of these intervals would contain the true parameter value.

In this article we provide an example of how a simple Bayesian analysis of a binary primary outcome could have been used instead of (or in addition to) traditional frequentist methods, to give a better insight into the clinical application of the results. We analyse a randomised controlled trial of two treatments for patients undergoing major gastrointestinal surgery, to arrive at a conclusion that may be more clinically interpretable and useful, and also provide a more positive message from the trial.

Methods

Data and Study Design

The Optimisation of Peri-operative Cardiovascular Management to Improve Surgical Outcome (OPTIMISE) trial [12] was a pragmatic, observer-blinded, multi-centre, randomised controlled trial of 734 high-risk patients (aged 50 years and older) that had undergone major gastrointestinal surgery in 17 acute care hospitals in the United Kingdom. Previous research suggested that postoperative outcomes may be improved by the use of cardiac output monitoring to guide the administration of intravenous fluid and inotropic drugs in the time around surgery [13]. Patients were randomly assigned (1:1) to a cardiac output-guided hemodynamic therapy algorithm for intravenous fluid and inotropic drug administration during and in the 6 hours following surgery (intervention; n=368) or to usual care (n=366). The primary outcome was a composite of predefined 30-day moderate or major complications and mortality.

The primary outcome occurred in 158/364 (43.41%) usual care and 134/366 (36.61%) intervention patients. At first glance, this would suggest that the results favour the intervention group compared with usual care. Using the traditional frequentist approach, the relative risk (RR) was found to be 0.84 (95% CI: 0.70, 1.01) and absolute risk reduction was 6.79% (95% CI: −0.30%, 13.89%), with p=0.07. This was interpreted as there being insufficient evidence that the RR for complication/death at 30 days is different to 1. Thus, the authors concluded, "use of this cardiac output–guided, hemodynamic therapy algorithm was not associated with a significant reduction in the composite primary outcome of moderate or major postoperative complications at 30 days following surgery." [12] A difference exists between the groups, but this was not judged to be sufficient using the conventional approach.

For peer review only - http://bmjopen.bmj.com/site/about/guidelines.xhtml

Patient involvement

Patients were not involved in the re-analysis of the OPTIMISE trial.

Statistical Methods

By repeating the same analysis using Bayesian methods we can find an alternative way to model and interpret these data. The Bayesian analysis involves specifying a prior distribution for the primary outcome rate in each treatment arm, and using the OPTIMISE trial data to update these prior distributions to become posterior distributions. From the posterior distributions we calculate the RR or absolute risk difference and 95% HDI. We also use the posterior distribution to calculate the probability that the intervention is superior (RR < 1 or absolute risk difference < 0).

We can extend this idea by specifying a clinically significant effect size. For instance, a group of clinicians or patients could decide that a RR must differ from the null by, say, 10% to be clinically significant. This “region of practical equivalence” (ROPE) can be used to make probability statements about the likely clinical significance of effect sizes. For illustrative purposes we specify the ROPEs to be 0.90 to 1.10 for the RR and -0.05 to 0.05 (-5% to 5%) for the absolute risk difference. These ROPEs may not be reasonable in practice, and should be developed based on clinician, patient and health economists’ feedback.

Additionally, we can simulate the outcomes of future patients using the updated estimates of the probabilities of having the primary outcome (from the posterior distribution) to predict the proportion of cases in which using the intervention would result in no complication/death whilst not using the intervention would.

We used two different types of prior distributions in separate analyses to check the robustness of our conclusions to our prior distribution assumptions. We used Beta distributions for the priors as these allowed the posterior distribution to be calculated more easily for our data. The sum of the two shape parameters in the Beta distribution provides an estimate of the effective sample size that the prior distribution provides, i.e. how much information it contributes.

Initially we used a flat prior for both arms, the Beta(1,1) distribution. This assumes that the prior distributions for the proportions for the composite outcome are uniform in each arm (see Figure 1, top panel), that is, all values between 0 and 1 are equally likely a priori. Whilst some values of the composite outcome rate are more likely than others, a flat prior may be used so that inferences from the posterior are driven by the trial data and probabilistic statements about the treatment effect can still be made. This prior contributes one additional patient with no complications and one patient with a complication/death. The results from this prior are unlikely to give different results to the original analysis, in terms of the RR and absolute risk difference, but the interpretations are likely to differ. A weakness of this approach is that too much prior probability is placed on extremely unlikely outcomes, i.e., the prior described gives an equal weighting to the probability of all patients having a complication and no patients having a complication.

When the results from the OPTIMISE trial were incorporated into an updated meta-analysis, it was found that the intervention reduced the incidence of 30-day complications/death following surgery: 31.52% (intervention) vs 41.60% (control), RR 0.77 (95% CI: 0.71, 0.83) [12]. We wanted to combine the previous information with the OPTIMISE trial data so that more precise treatment effect estimates could potentially be obtained and to pull the data away from inappropriate inferences.

Therefore, an evidence-based prior was also specified using the results from a pre-existing systematic review ([12, 13]) which had information on both treatment arms (Figures S1-S2, Supplementary Material). Figure 1 shows the evidence-based prior distributions for the control (middle panel) and intervention arm (bottom panel), where the distribution for the primary outcome rate in the intervention arm is centred at a lower value. Further details on how these priors were derived are displayed in the Supplementary Material. The evidence-based prior for the control arm contributed an effective sample size of 56 patients, and the intervention arm prior contributed 66 patients worth of information. With this additional information, the RR and absolute risk difference may decrease in favour of the intervention, compared to the original analyses.

The Bayesian analysis was conducted in R version 3.4.1 and additional information on the algorithm settings and the code for the Bayesian analysis is available in the Supplementary Material. Readers can perform the analysis here: https://argoshare.is.ed.ac.uk/bayesian_two_proportions/

[Figure 1 here]

Results

[Figure 2 here]

The results differ slightly between the flat and evidence-based priors, particularly for the probability of lying in the ROPE. Under the flat prior, the posterior mean RR is 0.85 (95% HDI: 0.70, 1.00), which is approximately the same as the frequentist analysis. Using the evidence-based priors, the posterior mean RR is 0.81 (95% HDI: 0.67, 0.95). The probability that the intervention group has a lower incidence of the composite endpoint is 96.9% and 99.50%, assuming a flat and evidence-based prior, respectively (Figure 2 (a) and (b)).

Based on the RR ROPE, the probability of the two arms being clinically equivalent was 24% and 9% under the flat and evidence-based priors, respectively. Alternatively, the probability of there being a clinically relevant difference in the RR of the composite endpoint between the two groups was 76% and 91% for the flat and evidence-based priors, respectively.

Using the flat prior, the posterior mean of the absolute risk difference/reduction was 6.76% (95% HDI: -0.30%, 13.80%), which is approximately the same as the frequentist analysis. Using the evidence-based prior, this value was 8.55% (95% HDI: 2.00%, 15.00%). When the ROPE was defined as an absolute risk difference of -5% to 5%, the probabilities of the trial arms being equivalent were 31% and 14% (69% and 86% probability of there being a clinically relevant difference) using the flat and evidence-based priors, respectively (see Figure 2 (c) and (d)).

The posterior probability of different RRs occurring is shown in Figure 3 (a) (flat prior) and (b) (evidence-based prior). For example, in Figure 3(a), the probability that the RR<1 is 0.97, and the probability RR<0.8 is 0.28.

Using the Bayesian model, predictions were obtained from the posterior distribution for future patients. These found that the probability that a patient in the intervention group did not have a complication/death when a patient in the control group did have a complication/death was 27.56% (flat prior) and 28.49% (evidence-based prior). (The probability of a patient in the intervention group

peer review only

having a complication/death when a patient in the control group did not was 20.77% and 19.59%, assuming a flat and evidence-based prior, respectively).

[Figure 3 here]

Discussion

In this work we have performed a Bayesian re-analysis of the OPTIMISE trial [12] to demonstrate how a Bayesian approach can more easily answer clinical questions of interest and inform decision making. Our conclusions from the analysis are given in terms of probabilities that a benefit exists. The Bayesian approach also enabled us to formally incorporate information from previous studies on the event rates for each arm and combine this information with the OPTIMISE trial data. The principal finding of this analysis was that the use of a Bayesian analytical approach provided a different interpretation of the findings of the OPTIMISE trial, suggesting patient benefit from the intervention.

The OPTIMISE trial was powered (at 90%) to detect a reduction in the primary outcome rate from 50% in the control group to 37.5% in the intervention group (RR 0.75, absolute risk reduction 12.5%). The frequentist analysis conducted by Pearse et al. [12] identified no significant difference for 30-day complications/death between the treatment arms and deemed the trial to be underpowered. The Bayesian posterior mean estimates and 95% HDI gave similar results to the frequentist analysis as expected, with more optimistic results produced by the evidence-based prior. (Previous evidence had suggested that the intervention was superior.) The primary advantage here of using the Bayesian approach is that we can explicitly obtain the posterior distribution of the primary outcome rates for each arm, and from this the treatment effect distribution, which can be interpreted directly and intuitively. From these distributions we can calculate the probability that the new treatment is superior. A frequentist analysis, particularly of an underpowered trial, provides little help for clinicians to make the necessary decisions regarding future treatment and also runs the risk of concluding no difference between treatments (based on a non-significant p-value), even when there is in reality a useful difference. By incorporating previous information into the prior distribution, the Bayesian approach can provide more informative probabilistic statements about the treatment effect.

For a reasonably large trial, different priors should have little effect on the posterior distributions as the data are contributing a large amount of information, and generally outweigh the contribution of the prior information. In this study, altering the priors had little impact on the results, most likely due to the fairly large sample size. The evidence-based prior used information from 21 small studies whose sample sizes ranged from 34 to 390 patients and were mostly in favour of the intervention. The evidence-based prior for the control arm contributed 56 patients worth of information compared to the 364 patients from OPTIMISE; the evidence-based prior for the intervention arm contributed 66 patients worth of information and the OPTIMISE trial contributed 366 patients. The analyses which used the evidence-based priors obtained a slightly smaller RR and provided more certainty that the intervention was superior, compared to the flat priors. Since the previous studies were small studies, one may wish to down weight the influence of these studies in the prior to consider the risk of bias. Plots of the priors overlaid with the posteriors for the evidence-based priors

For peer review only

are provided in the Supplementary Material (Figure S3) and show that the prior for the intervention was too optimistic since it is centred at a lower primary outcome rate than the posterior.

The Bayesian analyses that we have presented here are simple, and repeat the primary analysis of the OPTIMISE trial to demonstrate the usefulness of the Bayesian approach. These analyses could be extended to allow for more complicated models, such as Bayesian hierarchical models that account for site-specific effects or to allow for covariate adjustment. A Bayesian analysis cannot salvage a badly designed or conducted trial. Instead, the Bayesian approach can be used to produce results that are more easily interpreted and avoids the dichotomisations that occur in significance testing.

Conclusion

Using the results from a standard statistical analysis, the OPTIMISE trial authors concluded that the use of the intervention compared with usual care did not significantly reduce a composite outcome of 30-day complications and mortality [12]. Although the trial was not powered to detect a statistically significant difference between the treatment arms for the observed effect size, by using Bayesian analyses we were able to demonstrate that the intervention was superior to the control with 97-99.5% probability. These results are much more clinically useful than those that are given by the traditional approach.

References

[1] Simmons JP, Nelson LD, Simonsohn U. False-positive psychology: Undisclosed flexibility in data collection and analysis allows presenting anything as significant. Psychol Sci. 2011 Oct 17; 22(11):1359-1366.

[2] Greenland S, Senn SJ, Rothman KJ, Carlin JB, Poole CP, Goodman SN, Altman DG. Statistical tests, *P* values, confidence intervals, and power: a guide to misinterpretations. Eur J Epidemiol. 2016 May 21; 31: 337-350.

[3] Wasserstein RL, Lazar NA. The ASA's statement on p-values: context, process and purpose. The American Statistician. 2016 June 09; 70(2): 129-133

[4] McShane BB, Gal D, Gelman A, Robert C, Tackett JL. Abandon Statistical Significance. arXiv preprint arXiv:1709.07588. 2017 Sep 21.

[5] Alderson P, Chalmers I. Survey of claims of no effect in abstracts of Cochrane reviews. BMJ. 2003 Mar 01; 326(7387):475.

[6] Greenland S. Null misinterpretation in statistical testing and its impact on health risk assessment. Prev Med. 2011 Aug 17; 53:225–8.

[7] Altman DG, Bland JM. Statistics notes: Absence of evidence is not evidence of absence. BMJ. 1995 Aug 19; 311(7003):485.

For peer review only

[8] Trafimow D, Marks M. Editorial. Basic Appl. Soc. Psych. 2015 Feb 12; 37(1): 1-2

[9] Morris RK, Malin GL, Quinlan-Jones E, Middleton LJ, Hemming K, Burke D, et al. Percutaneous vesicoamniotic shunting versus conservative management for fetal lower urinary tract obstruction (PLUTO): a randomised trial. Lancet. 2013 Aug 13; 382(9903): 1496-1506.

[10] Laptook AR, Shankaran S, Tyson JE, Munoz B, Bell EF, Goldberg RN, et al. Effect of Therapeutic Hypothermia Initiated After 6 Hours of Age on Death or Disability Among Newborns With Hypoxic-Ischemic Encephalopathy A Randomized Clinical Trial. JAMA. 2017 October 31; 318(16): 1550-1560.

[11] Reardon MJ, Van Miegham NM, Popma JJ, Kleiman NS, Sondergaard L, Mumtaz M, et al. Surgical or Transcatheter Aortic-Valve Replacement in Intermediate-Risk Patients. NEJM. 2017 Mar 17; 376(14): 1321-1331.

[12] Pearse RM, Harrison DA, MacDonald N, Gillies MA, Blunt M, Ackland G, et al. Effect of a Perioperative, Cardiac Output–Guided Hemodynamic Therapy Algorithm on Outcomes Following Major Gastrointestinal Surgery A Randomized Clinical Trial and Systematic Review. JAMA. 2014 May 19; 311(21): 2181-2190.

[13] Grocott MP, Dushianthan A, Hamilton MA, Mythen MG, Harrison D, Rowan K; Optimisation Systematic Review Steering Group. Perioperative increase in global blood flow to explicit defined goals and outcomes following surgery. *Cochrane Database Syst Rev*. 2012 Nov 14;11:CD004082.

For peer review only

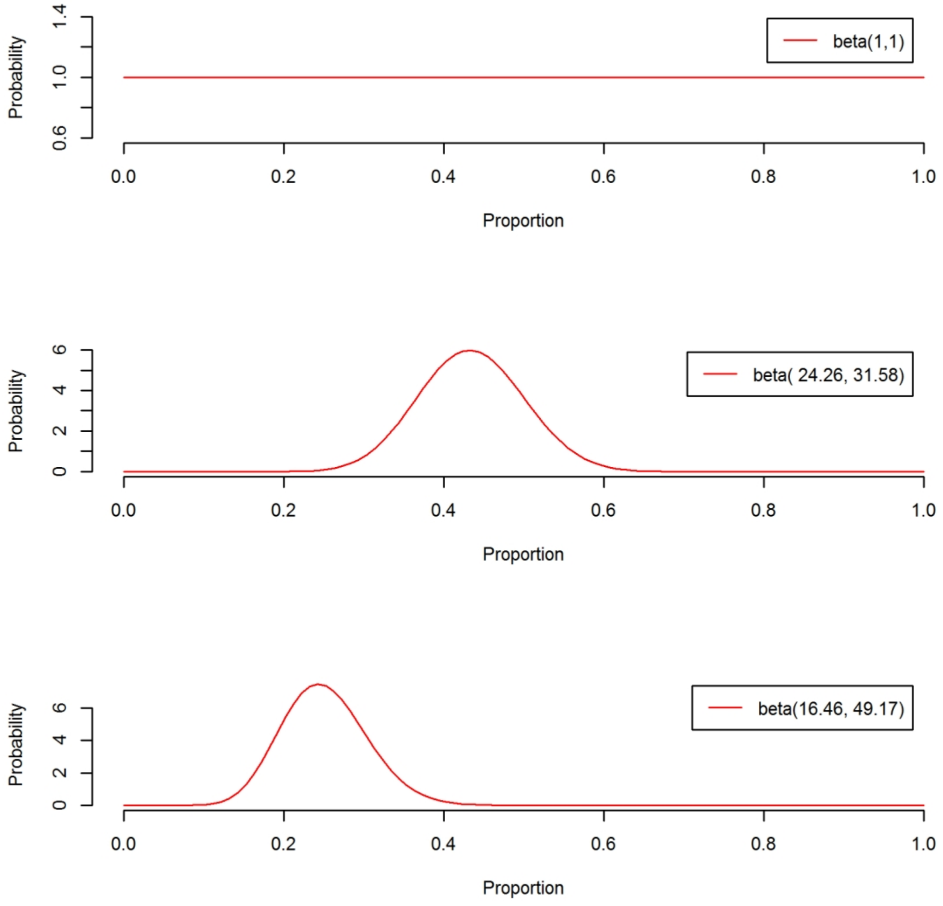

Figure 1. Prior distributions used in Bayesian reanalysis of OPTIMISE. The top panel is a flat prior distribution, beta(1,1), that was used for both treatment arms. The middle and bottom panels show the evidence-based priors that were derived using a meta-analysis. The middle panel is the prior for the control arm (beta(24.26, 31.58)) and the bottom panel is the prior for the intervention arm (beta(16.46, 49.17)).

152x152mm (300 x 300 DPI)

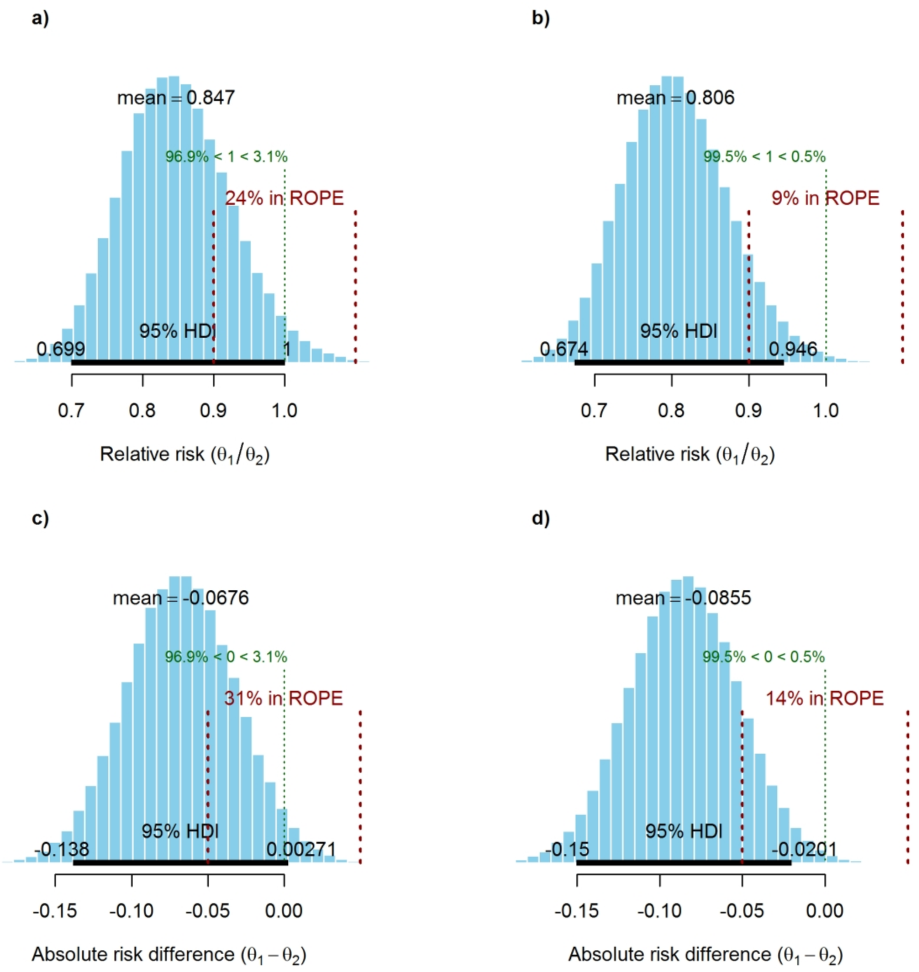

Figure 2. Posterior distributions of relative risk (RR) and absolute risk difference for the flat prior and evidence-based priors. θ1 and θ2 are the composite outcome rates in the intervention and control arms, respectively. Posterior distribution of the RR of the primary outcome using a) a flat prior, b) evidence-based priors. A RR>1 indicates that the intervention is more harmful. Posterior distribution of the absolute risk difference in the primary outcome using c) a flat prior d) evidence-based priors. A positive value of the absolute risk difference indicates that the intervention is more harmful. The 95% highest posterior density interval (HDI) is shown as the thick black horizontal line with the boundaries written above the line. The region of practical equivalence (ROPE) is shown in red (with dotted vertical lines). The posterior probability of RR<1 and RR>1, and an absolute difference <0 and >0 is shown in green above the ROPE

177x177mm (300 x 300 DPI)

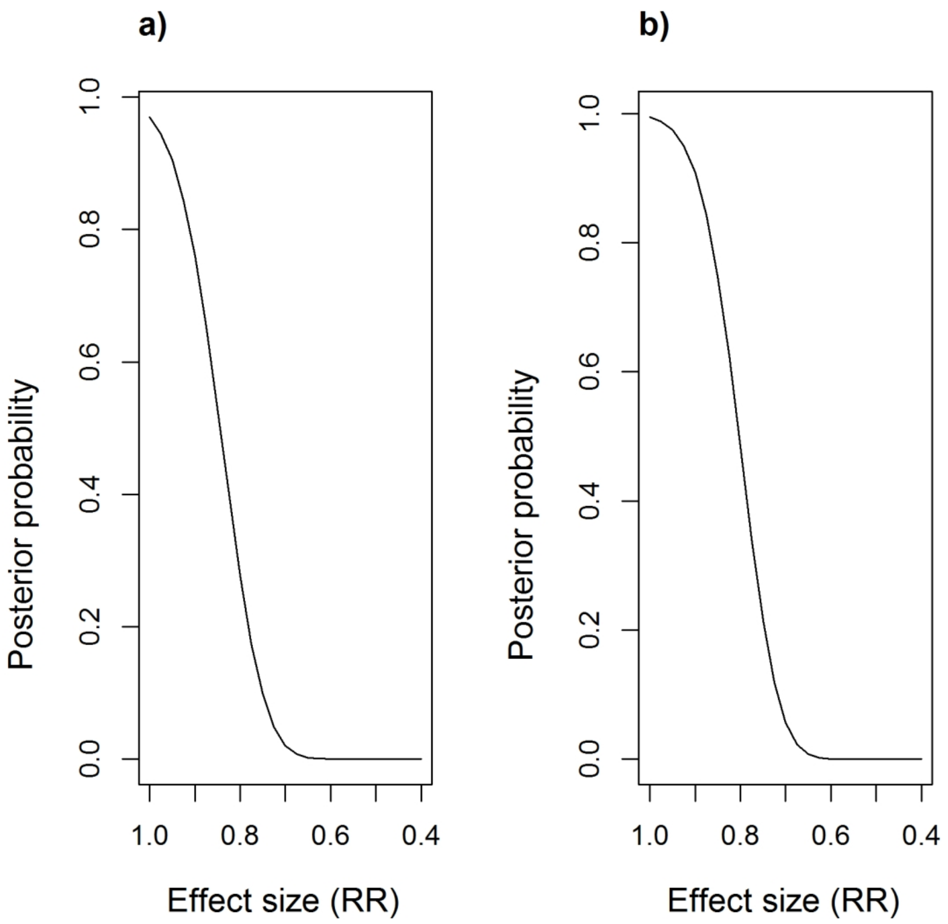

Figure 3. Posterior probability of specified effect sizes, using (a) flat prior, (b) evidence-based priors. The line shows the probability of the RR being lower than the values on the x-axis (i.e., a bigger treatment effect). A RR<1 indicates that the primary outcome rate is smaller in the intervention arm compared to the control arm

152x152mm (300 x 300 DPI)

Supplementary Material

Algorithms settings and code for Bayesian analysis

The Bayesian analysis was conducted in R version 3.4.1 using the *rjags* package [1], which provides an interface to the JAGS library (JAGS version 4.3.0) to perform Bayesian data analysis. JAGS (Just Another Gibbs Sampler) was derived from BUGS (Bayesian analysis Using Gibbs Sampling; see [2] for details on the BUGS project) and uses Markov Chain Monte Carlo (MCMC) to generate samples from the posterior distribution so that inference on the parameters of interest may be obtained. The posterior distribution was obtained using a sample of 200 000 estimates of the primary outcome probability for each arm (used 3 chains each of length 66667, with a burn-in period of 1000 iterations and 500 iterations were used to adapt/tune the samplers). Model convergence was checked using trace plots, density plots, and the Gelman-Rubin diagnostic [3]. The MCMC code is presented below in “R code 1”.

Since we used Beta distributions for the priors, and have binary data that is modelled by a binomial distribution, we are able to obtain an algebraic expression to calculate the posterior distribution. The Beta distribution prior is a *conjugate prior* for the binomial distribution, which means that the posterior distribution is in the same probability distribution family as the prior distribution (Beta distribution). We can update the Beta distribution parameters using the data from the trial:

$$Beta(\alpha_t + y_t, \beta_t + N_t - y_t)$$

where $\mathrm{Beta}(\alpha_{t,}, \beta_t)$ is the prior for treatment arm *t; y_t* are the number of complications/deaths at 30 days for arm t; and N_t are the number of patients randomised to arm t. We can simulate samples from this distribution for each arm (simulating from the posterior) and examine the relative risks (RR) and absolute risk differences. This code is presented in “R code 2”.

w only

R code 1. Code for posterior simulations using an MCMC algorithm

```
# OPTIMISE trial in a Bayesian framework

# JAMA. 2014;311(21):2181-2190. doi:10.1001/jama.2014.5305


remove(list=ls())


#specify your working directory

setwd("H:/Documents/optimise i")




# Primary outcome: composite of 30-day moderate or major complications and mortality

N1 <- 366

y1 <- 134
```

```
N2 <- 364

y2 <- 158

# N1 is total number in the Cardiac Output–Guided Hemodynamic Therapy Algorithm (intervention) group

# y1 is number with the outcome in the Cardiac Output–Guided Hemodynamic Therapy Algorithm (intervention) group

# N2 is total number in usual care (control) group

# y2 is number with the outcome in usual care (control) group


# Risk ratio

(y1/N1)/(y2/N2)


#frequentist analysis

library(epitools)

riskratio(c(N1-y1, y1, N2-y2, y2), rev="rows", method="boot", replicates=100000)


# Using standard frequentist approach

# Risk ratio (bootstrapped 95% confidence intervals) = 0.84 (0.70-1.01)

# p=0.07 (Fisher exact p-value)



#-----------------------------------------------------------------------------


# Bayesian analysis of same ratio

# Base script from Kruschke, J. K. (2011). Doing Bayesian Data Analysis, Academic Press / Elsevier.


#load required packages (install first if necessary)

require(rjags)

library(BEST)



# The model will be specified with uninformative prior distributions (beta(1,1,).


# Plot prior beta(1,1)

par(mfrow=c(1,1))

plot(seq(0, 1, length.out=100), dbeta(seq(0, 1, length.out=100), 1,1),

                    type="l", xlab="Proportion",

                    ylab="Probability",
```

For peer review only

```
                    main="OPTIMISE Composite Primary Outcome Prior distribution",
                    frame=FALSE, col="red", oma=c(6,6,6,6))
legend("topright", legend="beta(1,1)", lty=1, col="red", inset=0)

#------------------------------------------------------------------------------

# Specify the MODEL.
modelString = "
# JAGS model specification begins here...
model {
# Likelihood. Each complication/death is Bernoulli.
for ( i in 1 : N1 ) { y1[i] ~ dbern( theta1 ) }
for ( i in 1 : N2 ) { y2[i] ~ dbern( theta2 ) }
# Prior. Independent beta distributions. Change this for informative prior
theta1 ~ dbeta( 1,1) #intervention
theta2 ~ dbeta( 1,1) #control
}
# ... end JAGS model specification
" # close quote for modelstring

# Write the modelString to a file, using R commands:
writeLines(modelString,con="H:/Documents/optimise i/model.txt")



#------------------------------------------------------------------------------
# THE DATA.

# Specify the data in a form that is compatible with JAGS model, as a list:
dataList =        list(
         N1 = N1 ,
         y1 = c(rep(1, y1), rep(0, N1-y1)),
         N2 = N2 ,
         y2 = c(rep(1, y2), rep(0, N2-y2))
)

#------------------------------------------------------------------------------
```

For peer review only

```
# INTIALIZE THE CHAIN.

# Can be done automatically in jags.model() by commenting out inits argument.
# Otherwise could be established as:
# initsList = list( theta1 = sum(dataList$y1)/length(dataList$y1) ,
#                   theta2 = sum(dataList$y2)/length(dataList$y2) )

#------------------------------------------------------------------------------
# RUN THE CHAINS.

#specify the MCMC parameters
parameters = c( "theta1" , "theta2" )     # The parameter(s) to be monitored.
adaptSteps = 500              # Number of steps to "tune" the samplers.
burnInSteps = 1000            # Number of steps to "burn-in" the samplers.
nChains = 3                   # Number of chains to run.
numSavedSteps=200000          # Total number of steps in chains to save.
thinSteps=1                   # Number of steps to "thin" (1=keep every step).
nIter = ceiling( ( numSavedSteps * thinSteps ) / nChains ) # Steps per chain.



# Create, initialize, and adapt the model:
jagsModel = jags.model( "H:/Documents/optimise i/model.txt" , data=dataList , # inits=initsList ,
                        n.chains=nChains , n.adapt=adaptSteps )
# Burn-in:
cat( "Burning in the MCMC chain...n" )

#run the MCMC
update( jagsModel , n.iter=burnInSteps )

# The saved MCMC chain:
cat( "Sampling final MCMC chain...n" )
codaSamples = coda.samples( jagsModel , variable.names=parameters ,
                        n.iter=nIter , thin=thinSteps )

# resulting codaSamples object has these indices:
```

For peer review only

```
#   codaSamples[[ chainIdx ]][ stepIdx , paramIdx ]



#check MCMC has converged

plot(codaSamples)

summary(window(codaSamples, start = burnInSteps))

# Gelman-Rubin diagostic - factor of 1 means that between chain variance and within chain variance are equal.
Values <=1.1 suggest convergence

gelman.diag(codaSamples)

#Check whether the low chain reduction is stable.

gelman.plot(codaSamples)

#Burn-in appear sufficient (choose the iteration after which the lines stay below 1.1)



#------------------------------------------------------------------------------

# EXAMINE THE RESULTS.


# Convert coda-object codaSamples to matrix object for easier handling.

# This concatenates the different chains into one long chain.

# Result is mcmcChain[ stepIdx , paramIdx ]

mcmcChain = as.matrix( codaSamples )



theta1Sample = mcmcChain[,"theta1"] # Put sampled values in a vector - INTERVENTION ARM.

theta2Sample = mcmcChain[,"theta2"] # Put sampled values in a vector - CONTROL ARM




# Plot a histogram of the posterior differences of theta values.

thetaRR = theta1Sample / theta2Sample # Relative risk

thetaDiff = theta1Sample - theta2Sample # Absolute risk difference


# 2x1 plots

par(mfrow=c(2,1))


# plot posterior of RR

par(mar=c(4, 4.1, 4.1, 4))
```

For peer review only

```
plotPost( thetaRR , xlab= expression(paste("Relative risk (", theta[1]/theta[2], ")")) ,
        compVal=1.0, ROPE=c(0.9, 1.1),
        main="OPTIMISE Composite Primary Outcome
Posterior distribution of relative risk", cex.main=1, cex=1, cex.lab=1, xlab=c(0.6,1.4))

#Plot absolute risk difference
plotPost( thetaDiff , xlab=expression(paste("Absolute risk difference (", theta[1]-theta[2], ")")) ,
        compVal=0.0, ROPE=c(-0.05, 0.05),
        main="OPTIMISE Composite Primary Outcome
Posterior distribution of absolute risk difference", cex.main=1, cex=1, cex.lab=1, xlab=c(-0.25, 0.15))



#------------------------------------------------------------------------------

# Use posterior prediction to determine proportion of cases in which
# using the intervention would result in no complication/death
# while not using the intervention would result in complication death

chainLength = length( theta1Sample )

# Create matrix to hold results of simulated patients:
yPred = matrix( NA , nrow=2 , ncol=chainLength )

# For each step in chain, use posterior prediction to determine outcome
for ( stepIdx in 1:chainLength ) { # step through the chain
        # Probability for complication/death for each "patient" in intervention group:
        pDeath1 = theta1Sample[stepIdx]
        # Simulated outcome for each intervention "patient"
        yPred[1,stepIdx] = sample( x=c(0,1), prob=c(1-pDeath1,pDeath1), size=1 )
        # Probability for complication/death for each "patient" in control group:
        pDeath2 = theta2Sample[stepIdx]
        # Simulated outcome for each control "patient"
        yPred[2,stepIdx] = sample( x=c(0,1), prob=c(1-pDeath2,pDeath2), size=1 )
}
```

For peer review only

```
# Now determine the proportion of times that the intervention group has no complication/death
# (y1 == 0) and the control group does have a complication or death (y2 == 1))
(pY1eq0andY2eq1 = sum( yPred[1,]==0 & yPred[2,]==1 ) / chainLength)

#proportion of times intervention has complication/death where control group does not
(pY1eq1andY2eq0 = sum( yPred[1,]==1 & yPred[2,]==0 ) / chainLength)

#proportion of times where neither arm has complication/death
(pY1eq0andY2eq0 = sum( yPred[1,]==0 & yPred[2,]==0 ) / chainLength)

#proportion of times where both arms have complication/death
(pY10eq1andY2eq1 = sum( yPred[1,]==1 & yPred[2,]==1 ) / chainLength)
```

For peer review only

R Code 2. Code for exact posterior calculation

```
remove(list=ls())

###uninformative prior

#prior for control group is beta(a1, b1)
a1=1
b1=1

#prior for treatment group is beta(a2, b2)
a2=1
b2=1

#control event/total is z1/N1
z1=158
N1=364

#treatment event/total is z2/N2.
z2=134
N2=366

#simulate from the posterior for control group. posterior can be calculated exactly as beta(a1+z1, b1+N1-z1)
pc = rbeta(200000, a1+z1, b1+N1-z1)
#simulate from the posterior for intervention group. posterior can be calculated exactly as beta(a2+z2, b2+N2-z2)
pt = rbeta(200000, a2+z2, b2+N2-z2)


thetaRR = pt / pc # Relative risk
thetaDiff = pt - pc # Absolute risk difference

############################################################################
############################################################################
#evidence-based prior

#prior for control group is beta(a1, b1)
a1=24.26
b1=31.58


#prior for treatment group is beta(a2, b2)
a2=16.46
b2=49.17

#control event/total is z1/N1
z1=158
N1=364

#treatment event/total is z2/N2.
z2=134
N2=366

#simulate from the posterior for control group. posterior can be calculated exactly as beta(a1+z1, b1+N1-z1)
```

For peer review only

```
pcc = rbeta(200000, a1+z1, b1+N1-z1)
#simulate from the posterior for intervention group. posterior can be calculated exactly as beta(a2+z2, b2+N2-z2)
ptt = rbeta(200000, a2+z2, b2+N2-z2)



thetaRR_ev = ptt / pcc # Relative risk
thetaDiff_ev = ptt - pcc # Absolute risk difference


###########################################################################

#plot posterior distributions

require(rjags)
library(BEST)

# 2x2 plots
par(mfrow=c(2,2))

# plot posterior for uninformative prior, RR
par(mar=c(4, 4.1, 4.1, 4))
plotPost( thetaRR , xlab= expression(paste("        Relative risk (", theta[1]/theta[2], ")")) ,
        compVal=1.0, ROPE=c(0.9, 1.1),
        main="a)", adj=0,cex.main=1, cex=1, cex.lab=1, xlab=c(0.6,1.4))

# plot posterior for uninformative prior, absolute risk difference
plotPost( thetaDiff , xlab=expression(paste("Absolute risk difference (", theta[1]-theta[2], ")")) ,
        compVal=0.0, ROPE=c(-0.05, 0.05),
        main="b)", adj=0,cex.main=1, cex=1, cex.lab=1, xlab=c(-0.25, 0.15))

# plot posterior for evidence-based prior, RR
par(mar=c(4, 4.1, 4.1, 4))
plotPost( thetaRR_ev , xlab= expression(paste("        Relative risk (", theta[1]/theta[2], ")")) ,
        compVal=1.0, ROPE=c(0.9, 1.1),
        main="c)", adj=0,cex.main=1, cex=1, cex.lab=1, xlab=c(0.6,1.4))

# plot posterior for evidence-based prior, absolute risk difference
plotPost( thetaDiff_ev , xlab=expression(paste("Absolute risk difference (", theta[1]-theta[2], ")")) ,
        compVal=0.0, ROPE=c(-0.05, 0.05),
        main="d)", adj=0,cex.main=1, cex=1, cex.lab=1, xlab=c(-0.25, 0.15))
```

For peer review only

Evidence-based Prior

An evidence-based prior was specified using results from a pre-existing meta-analysis ([4,5] – not including the OPTIMISE results) which had information on both treatment arms. There is no clear best approach of how to include historical information into the analysis of a clinical trial. Viele et al. [6] provide a good overview of the available approaches when data for the control arm is available and discuss how to decide to what extent historical data should be incorporated.

The R Bayesian evidence synthesis Tools (RBesT; [7]) were used to perform a Bayesian meta-analysis, which accounts for the uncertainty of the population mean and between-trial heterogeneity (using the *gMAP* function in R (version 3.4.1)). We used this approach as it is a principled and reproducible method of combining data from previous trials. Further refinements could also be included, such as attempting to correct for publication or other biases. The meta-analysis of the control and intervention arms are displayed in Figures S1 and S2, respectively.

For the control arm, the meta-analysis gave a mean estimate of 0.432 (95% HDI: 0.306, 0.563). Using the *betaExpert* function (from the *prevalence* package [8]) in R version 3.4.1 (setting the mean from the meta-analysis to the mode of the beta distribution, and using the 95% HDI) gave a beta(24.26,31.58) distribution. This was used as the prior for the control arm.

For the intervention arm, the meta-analysis gave a mean estimate of 0.243 (95% HDI: 0.148, 0.358). Using the *betaExpert* function [8] in R (setting the mean from the meta-analysis to the mode of the beta distribution, and using the 95% HDI) gave a beta(16.46, 49.17) distribution. This was used as the prior for the intervention arm.

We note that power priors [9] could have been used instead for deriving the evidence-based priors, which incorporate a weight parameter that determines how much information is used from the historical data.

For peer review only

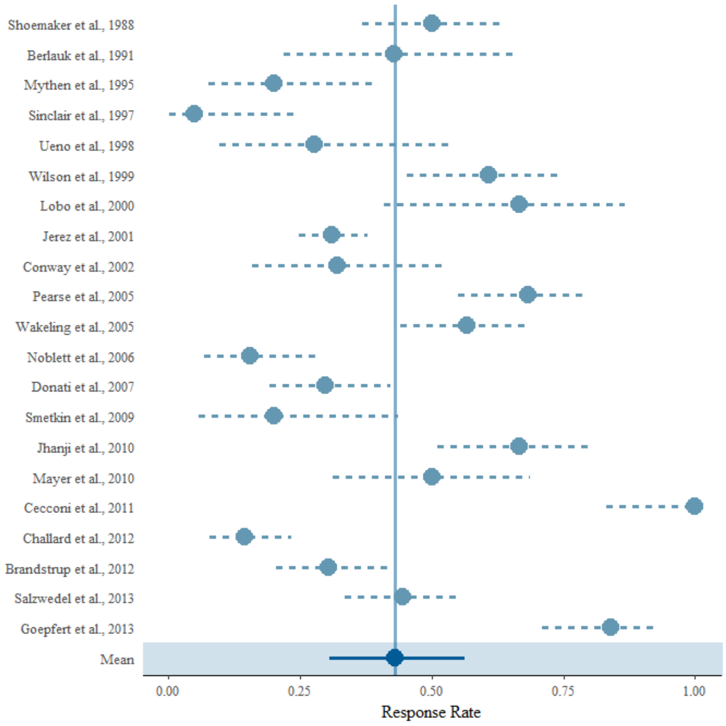

Figure S1.Forest plot of control arm using meta-analysis from Pearse et al. [5]. Dashed lines represent the stratified estimates with 95% HDI. Solid line represents the mean estimate (with 95% HDI))

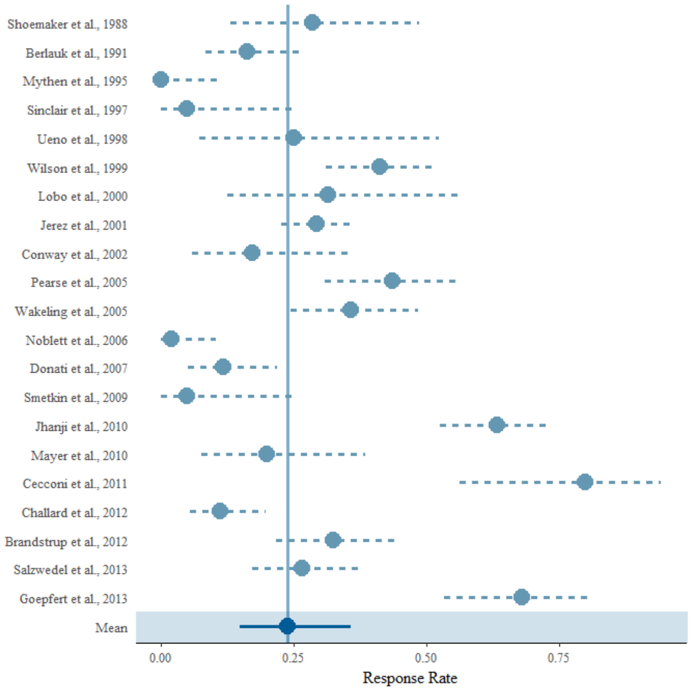

Figure S2. Forest plot of intervention arm using meta-analysis from Pearse et al. [5]. Dashed lines represent the stratified estimates with 95% HDI. Solid line represents the mean estimate (with 95% HDI))

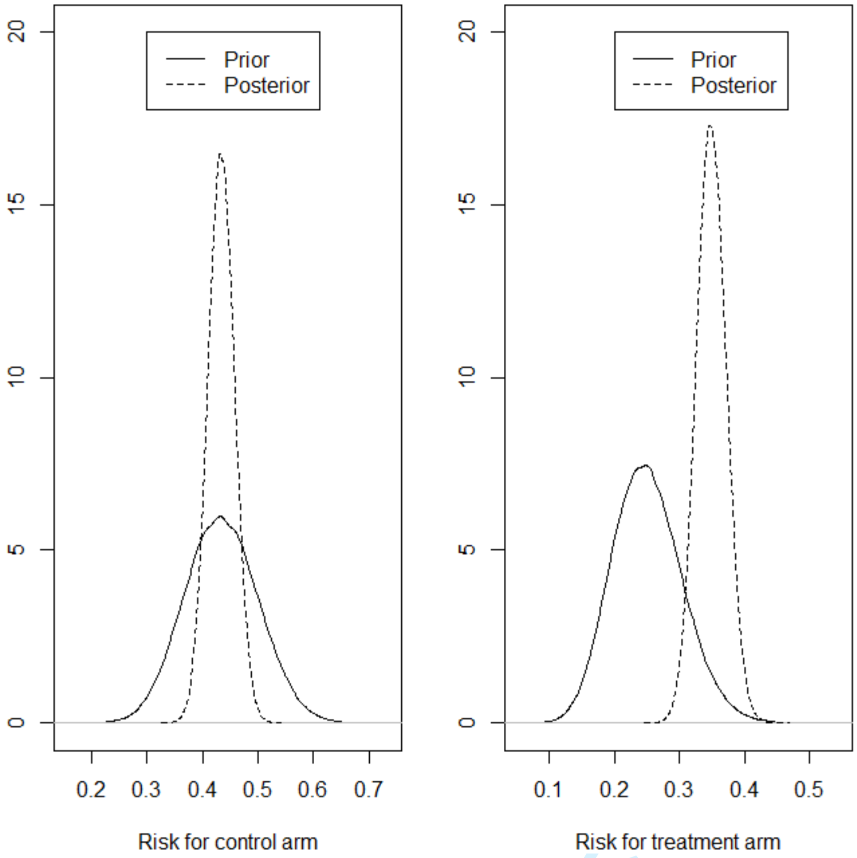

Figure S3. Plot of the evidence-based priors overlaid with the posteriors for each treatment arm

References

[1] Plummer M. JAGS: A program for analysis of Bayesian graphical models using Gibbs sampling. Proceedings of the 3rd International Workshop on Distributed Statistical Computing (DSC 2003). March 2003. p. 20–2.

[2] Lunn D, Spiegelhalter D, Thomas A, Best N. The BUGS project: Evolution, critique and future directions. Stat Med. 2009 October 13; 28(25): 3049-3067

[3] Gelman A, Rubin DB. Inference from Iterative Simulation Using Multiple Sequences. Stat Sci. 1992; 7(4): 457-472.

[4] Grocott MP, Dushianthan A, Hamilton MA, Mythen MG, Harrison D, Rowan K; Optimisation Systematic Review Steering Group. Perioperative increase in global blood flow to explicit defined goals and outcomes following surgery. *Cochrane Database Syst Rev*. 2012 Nov 14;11:CD004082.

[5] Pearse RM, Harrison DA, MacDonald N, Gillies MA, Blunt M, Ackland G, et al. Effect of a Perioperative, Cardiac Output–Guided Hemodynamic Therapy Algorithm on Outcomes

view only

Following Major Gastrointestinal Surgery A Randomized Clinical Trial and Systematic Review. JAMA. 2014 May 19; 311(21): 2181-2190.

[6] Viele K, Berry S, Neuenschwander B, Amzal B, Chen F, Enas N, et al. Use of historical control data for assessing treatment effects in clinical trials. Pharm Stat. 2013 August 5; 13:41-54

[7] Weber S. (2017). RBesT: R Bayesian Evidence Synthesis Tools. R package version 1.2-3. https://CRAN.R-project.org/package=RBesT

[8] Devleesschauwer B, Torgerson P, Charlier J, Levecke B, Praet N, Roelandt S, et al. (2014). prevalence: Tools for prevalence assessment studies. R package version 0.4.0. http://cran.r-project.org/package=prevalence

[9] Chen M-H, Ibrahim JG, Shao Q-M. Power prior distributions for generalized linear models. J Stat Plan Infer. 2000 Mar; 84(1-2):121-137

For peer review only

BMJ Open

Perioperative haemodynamic therapy for major gastrointestinal surgery: The effect of a Bayesian approach to interpreting the findings of a randomised controlled trial

Journal:	*BMJ Open*
Manuscript ID	bmjopen-2018-024256.R2
Article Type:	Research
Date Submitted by the Author:	30-Jan-2019
Complete List of Authors:	Ryan, Elizabeth; University of Warwick, Warwick Clinical Trials Unit; University of Birmingham, Cancer Research UK Clinical Trials Unit<br>Harrison, Ewen; Univeristy of Edinburgh, Clinical Surgery<br>Pearse, Rupert; Queen Mary University of London, Barts and the London School of Medicine & Dentistry<br>Gates, Simon; University of Birmingham, Cancer Research UK Clinical Trials Unit; University of Warwick, Warwick Clinical Trials Unit
**Primary Subject Heading**:	Research methods
Secondary Subject Heading:	Gastroenterology and hepatology, Surgery
Keywords:	Bayesian analysis, randomised controlled trial, SURGERY, peri-operative care

SCHOLARONE™
Manuscripts

Perioperative haemodynamic therapy for major gastrointestinal surgery: The effect of a Bayesian approach to interpreting the findings of a randomised controlled trial

Elizabeth G. Ryan*†, Ewen M. Harrison*, Rupert M. Pearse, and Simon Gates

***joint first authors**

†Corresponding author
Dr Elizabeth G. Ryan
Cancer Research Clinical Trials Unit, Institute of Cancer and Genomic Sciences, University of Birmingham, Birmingham, United Kingdom B15 2TT
phone: (+44) 0121 414 3289
Email: mailto:E.G.Ryan@bham.ac.uk

Email addresses:
EGR: E.G.Ryan@bham.ac.uk
EMH: mail@ewenharrison.com
RMP: r.pearse@qmul.ac.uk
SG: S.Gates@bham.ac.uk

Abstract

Objective: The traditional approach of null hypothesis testing dominates the design and analysis of randomised controlled trials. This study aimed to demonstrate how a simple Bayesian analysis could have been used to analyse the OPTIMISE trial to obtain more clinically interpretable results.

Design, setting, participants, and interventions: The OPTIMISE trial was a pragmatic, multicentre, observed-blinded, randomised controlled trial of 734 high-risk patients undergoing major gastrointestinal surgery in 17 acute care hospitals in the United Kingdom. Patients were randomly allocated to a cardiac output-guided hemodynamic therapy algorithm for intravenous fluid and inotropic drug administration during and in the 6 hours following surgery (n=368) or to standard care (n=366). The primary outcome was a binary outcome consisting of a composite of predefined 30-day moderate or major complications and mortality.

Methods: We repeated the primary outcome analysis of the OPTIMISE trial using Bayesian statistical methods to calculate the probability that the intervention was superior, and the probability that a clinically relevant difference existed. We explored the impact of a flat prior and an evidence-based prior on our analyses.

Results: Although OPTIMISE was not powered to detect a statistically significant difference between the treatment arms for the observed effect size (RR=0.84, 95% CI: 0.70, 1.01; p=0.07), by using Bayesian analyses we were able to demonstrate that there was a 96.9% (flat prior) to 99.5% (evidence-based prior) probability that the intervention was superior to the control.

For peer review only

Conclusions: The use of a Bayesian analytical approach provided a different interpretation of the findings of the OPTIMISE trial (compared to the original frequentist analysis), and suggested patient benefit from the intervention. Incorporation of information from previous studies provided further evidence of a benefit from the intervention. Bayesian analyses can produce results that are more easily interpretable and relevant to clinicians and policy makers.

Trial registration: isrctn.org Identifier: ISRCTN04386758

Keywords: Bayesian analysis; randomised controlled trial; surgery; peri-operative care

Word count: 2914

For peer review only

Strengths and limitations

- The use of a Bayesian analytical approach provided a different interpretation of the findings of the OPTIMISE trial (compared to the original frequentist analysis).
- The Bayesian approach avoids the dichotomisation of results that occur in significance testing.
- Bayesian analyses can produce results that are more easily interpretable and relevant to clinicians and policy makers.
- The analysis only used simple Bayesian methods and more sophisticated approaches can be used, such as adjusting for covariates.

Funding: EGR and SG were supported by a Medical Research Council (MRC) Methodology Research Grant (Grant number: MR/N028287/1).

Competing Interests: RMP holds research grants, and has given lectures and/or performed consultancy work for Nestle Health Sciences, BBraun, Medtronic, Glaxo Smithkline, Intersurgical, and Edwards Lifesciences, and is a member of the Associate editorial board of the British Journal of Anaesthesia.

Author Contributions: EMH and EGR wrote the statistical code and performed the Bayesian analysis, interpreted the findings and wrote the manuscript; SG contributed to the writing of the manuscript; RMP was the CI for the OPTIMISE study, was the lead author on the primary OPTIMISE paper, and contributed to the writing of the manuscript.

Patient consent: Written informed consent was obtained from all patients prior to surgery in the OPTIMISE trial.

Ethical approval: OPTIMISE was approved by the East London and City Research Ethics Committee and the Medical and Healthcare Products Regulatory Agency.

Data sharing agreement: Statistical code is available in the Supplementary Material.

Introduction

The traditional statistical approach of null hypothesis testing dominates clinical trial design and analysis. In this *frequentist* framework, conclusions are drawn using *p*-values and confidence intervals which have been generated to test a specific hypothesis (usually a null hypothesis of exactly zero treatment difference). It requires that a correct conclusion be drawn with a high level of probability (statistical power) from a notional set of repetitions of the trial.

The problems with this approach are well described (e.g., [1-4]). Frequentist results (*p*-values and confidence intervals) do not have simple or intuitive interpretations, and have often been misused and misinterpreted in clinical trials (see [2]). Randomised controlled trial results are often divided into “significant” and “non-significant”, usually based on a *p*-value < 0.05. A statistically non-significant result for the primary endpoint is generally interpreted as the new treatment being “unsuccessful” (see [5, 6]). This is often an incorrect interpretation, and was highlighted by Altman and Bland [7], “absence of evidence is not evidence of absence”. Randomised controlled trials are regarded as the gold standard of evidence and their results often inform clinical guidelines and practice. The misuse and misinterpretation of *p*-values (and confidence intervals) may result in the abandonment of potentially beneficial treatments. McShane et al. [4] recommended that null hypothesis testing should be abandoned altogether, and one peer-reviewed journal has banned its use [8].

Bayesian statistical methods provide an alternative framework for statistical modelling, and are increasingly being used in randomised controlled trials (e.g., [9-11]). The Bayesian approach can be used at the design stage and for data analysis. Bayesian statistics provide a formal method for combining pre-existing information with data that is collected in the clinical trial into the analysis so that the current state of knowledge can be updated. This is particularly useful for clinical research given that advances in health care usually occur through incremental gains in knowledge. The Bayesian approach may also provide results that are easier to interpret than the frequentist approach since it computes the probability of various values of the treatment effect, given the data.

Pre-existing information is incorporated into the analysis via a *prior distribution*. This is a probability distribution that accounts for uncertainty in an unknown parameter, for example, the treatment effect, before the data from the clinical trial has been incorporated. The frequentist approach assumes the parameter to have a fixed, but unknown value. Some are put off by this aspect of the Bayesian approach, expressing a concern that it is not scientifically objective. However, any statistical method, including traditional frequentist methods, involve subjective choices. For instance, the arbitrarily set 5% significance level in the frequentist approach. Moreover, so-called *non-informative priors* can be used when there is little reliable previous information, or when one would prefer to numerically mimic a frequentist analysis and avoid introducing external information into the analysis. These are often used as a default prior.

When clinical trial data are observed, the prior distribution is updated to become the *posterior distribution*, which summarises the current knowledge. The posterior distribution of the treatment effect provides the relative credibility of the range of treatment effect values, given the trial data. Calculation of the posterior distribution can often be straightforward, however, many practical examples require computationally intensive approaches. Part of the reason Bayesian statistics is becoming more popular is due to the advances in computing power and computational techniques. The posterior distribution can be used to answer questions of direct relevance to decision-making, such as “What is the probability that the new treatment is more successful than the current

For peer review only

treatment?" Frequentist methods are limited as to what research questions they can answer, and cannot directly provide answers to clinically important questions.

Rather than using confidence intervals (CIs), Bayesian statistics can use credible intervals, which provide a range of values for the treatment effect for a certain level of posterior probability. The highest posterior density interval (HDI) is the narrowest type of credible interval available for a specified probability. Although frequentist CI and Bayesian HDI may sometimes be numerically similar, their interpretation is different. A 95% HDI says, given the observed data, there is a 95% probability that the true parameter value falls within this interval. The frequentist interpretation of a 95% CI says, if this trial were to be repeated many times and CIs were calculated, 95% of these intervals would contain the true parameter value.

In this article we provide an example of how a simple Bayesian analysis of a binary primary outcome could have been used instead of (or in addition to) traditional frequentist methods, to give a better insight into the clinical application of the results. We analyse a randomised controlled trial of two treatments for patients undergoing major gastrointestinal surgery, to arrive at a conclusion that may be more clinically interpretable and useful, and also provide a more positive message from the trial.

Methods

Data and Study Design

The Optimisation of Peri-operative Cardiovascular Management to Improve Surgical Outcome (OPTIMISE) trial [12] was a pragmatic, observer-blinded, multi-centre, randomised controlled trial of 734 high-risk patients (aged 50 years and older) that had undergone major gastrointestinal surgery in 17 acute care hospitals in the United Kingdom. Previous research suggested that postoperative outcomes may be improved by the use of cardiac output monitoring to guide the administration of intravenous fluid and inotropic drugs in the time around surgery [13]. Patients were randomly assigned (1:1) to a cardiac output-guided hemodynamic therapy algorithm for intravenous fluid and inotropic drug administration during and in the 6 hours following surgery (intervention; n=368) or to usual care (n=366). The primary outcome was a composite of predefined 30-day moderate or major complications and mortality.

The primary outcome occurred in 158/364 (43.41%) usual care and 134/366 (36.61%) intervention patients. At first glance, this would suggest that the results favour the intervention group compared with usual care. Using the traditional frequentist approach, the relative risk (RR) was found to be 0.84 (95% CI: 0.7, 1.01) and absolute risk reduction was 6.79% (95% CI: −0.30%, 13.89%), with p=0.07. This was interpreted as there being insufficient evidence that the RR for complication/death at 30 days is different to 1. Thus, the authors concluded, "use of this cardiac output–guided, hemodynamic therapy algorithm was not associated with a significant reduction in the composite primary outcome of moderate or major postoperative complications at 30 days following surgery." [12] A difference exists between the groups, but this was not judged to be sufficient using the conventional approach.

For peer review only - http://bmjopen.bmj.com/site/about/guidelines.xhtml

Patient involvement

Patients were not involved in the re-analysis of the OPTIMISE trial.

Statistical Methods

By repeating the same analysis using Bayesian methods we can find an alternative way to model and interpret these data. The Bayesian analysis involves specifying a prior distribution for the primary outcome rate in each treatment arm, and using the OPTIMISE trial data to update these prior distributions to become posterior distributions. From the posterior distributions we calculate the RR or absolute risk difference and 95% HDI. We also use the posterior distribution to calculate the probability that the intervention is superior (RR < 1 or absolute risk difference < 0).

We can extend this idea by specifying a clinically significant effect size. For instance, a group of clinicians or patients could decide that a RR must differ from the null by, say, 10% to be clinically significant. This "region of practical equivalence" (ROPE) can be used to make probability statements about the likely clinical significance of effect sizes. For illustrative purposes we specify the ROPEs to be 0.9 to 1.1 for the RR and -0.05 to 0.05 (-5% to 5%) for the absolute risk difference. These ROPEs may not be reasonable in practice, and should be developed based on clinician, patient and health economists' feedback.

Additionally, we can simulate the outcomes of future patients using the updated estimates of the probabilities of having the primary outcome (from the posterior distribution) to predict the proportion of cases in which using the intervention would result in no complication/death whilst not using the intervention would.

We used two different types of prior distributions in separate analyses to check the robustness of our conclusions to our prior distribution assumptions. We used Beta distributions for the priors as these allowed the posterior distribution to be calculated more easily for our data. The sum of the two shape parameters in the Beta distribution provides an estimate of the effective sample size that the prior distribution provides, i.e. how much information it contributes.

Initially we used a flat prior for both arms, the Beta(1,1) distribution. This assumes that the prior distributions for the proportions for the composite outcome are uniform in each arm (see Figure 1, top panel), that is, all values between 0 and 1 are equally likely a priori. Whilst some values of the composite outcome rate are more likely than others, a flat prior may be used so that inferences from the posterior are driven by the trial data and probabilistic statements about the treatment effect can still be made. This prior contributes one additional patient with no complications and one patient with a complication/death. The results from this prior are unlikely to give different results to the original analysis, in terms of the RR and absolute risk difference, but the interpretations are likely to differ. A weakness of this approach is that too much prior probability is placed on extremely unlikely outcomes, i.e., the prior described gives an equal weighting to the probability of all patients having a complication and no patients having a complication.

When the results from the OPTIMISE trial were incorporated into an updated meta-analysis, it was found that the intervention reduced the incidence of 30-day complications/death following surgery: 31.52% (intervention) vs 41.60% (control), RR 0.77 (95% CI: 0.71, 0.83) [12]. We wanted to combine the previous information with the OPTIMISE trial data so that more precise treatment effect estimates could potentially be obtained and to pull the data away from inappropriate inferences.

Therefore, an evidence-based prior was also specified using the results from a pre-existing systematic review ([12, 13]) which had information on both treatment arms (Figures S1-S2, Supplementary Material). Figure 1 shows the evidence-based prior distributions for the control (middle panel) and intervention arm (bottom panel), where the distribution for the primary outcome rate in the intervention arm is centred at a lower value. Further details on how these priors were derived are displayed in the Supplementary Material. The evidence-based prior for the control arm contributed an effective sample size of 56 patients, and the intervention arm prior contributed 66 patients worth of information. With this additional information, the RR and absolute risk difference may decrease in favour of the intervention, compared to the original analyses.

The Bayesian analysis was conducted in R version 3.4.1 and additional information on the algorithm settings and the code for the Bayesian analysis is available in the Supplementary Material. Readers can perform the analysis here: https://argoshare.is.ed.ac.uk/bayesian_two_proportions/

[Figure 1 here]

Results

[Figure 2 here]

The results differ slightly between the flat and evidence-based priors, particularly for the probability of lying in the ROPE. Under the flat prior, the posterior mean RR is 0.85 (95% HDI: 0.70, 1.00), which is approximately the same as the frequentist analysis. Using the evidence-based priors, the posterior mean RR is 0.81 (95% HDI: 0.67, 0.95). The probability that the intervention group has a lower incidence of the composite endpoint is 96.9% and 99.5%, assuming a flat and evidence-based prior, respectively (Figure 2 (a) and (b)).

Based on the RR ROPE, the probability of the two arms being clinically equivalent was 24% and 9% under the flat and evidence-based priors, respectively. Alternatively, the probability of there being a clinically relevant difference in the RR of the composite endpoint between the two groups was 76% and 91% for the flat and evidence-based priors, respectively.

Using the flat prior, the posterior mean of the absolute risk difference/reduction was 6.76% (95% HDI: -0.30%, 13.80%), which is approximately the same as the frequentist analysis. Using the evidence-based prior, this value was 8.55% (95% HDI: 2.00%, 15.00%). When the ROPE was defined as an absolute risk difference of -5% to 5%, the probabilities of the trial arms being equivalent were 31% and 14% (69% and 86% probability of there being a clinically relevant difference) using the flat and evidence-based priors, respectively (see Figure 2 (c) and (d)).

The posterior probability of different RRs occurring is shown in Figure 3 (a) (flat prior) and (b) (evidence-based prior). For example, in Figure 3(a), the probability that the RR<1 is 0.97, and the probability RR<0.8 is 0.28.

Using the Bayesian model, predictions were obtained from the posterior distribution for future patients. These found that the probability that a patient in the intervention group did not have a complication/death when a patient in the control group did have a complication/death was 27.56% (flat prior) and 28.49% (evidence-based prior). (The probability of a patient in the intervention group

For peer review only

having a complication/death when a patient in the control group did not was 20.77% and 19.59%, assuming a flat and evidence-based prior, respectively).

[Figure 3 here]

Discussion

In this work we have performed a Bayesian re-analysis of the OPTIMISE trial [12] to demonstrate how a Bayesian approach can more easily answer clinical questions of interest and inform decision making. Our conclusions from the analysis are given in terms of probabilities that a benefit exists. The Bayesian approach also enabled us to formally incorporate information from previous studies on the event rates for each arm and combine this information with the OPTIMISE trial data. The principal finding of this analysis was that the use of a Bayesian analytical approach provided a different interpretation of the findings of the OPTIMISE trial, suggesting patient benefit from the intervention.

The OPTIMISE trial was powered (at 90%) to detect a reduction in the primary outcome rate from 50% in the control group to 37.5% in the intervention group (RR 0.75, absolute risk reduction 12.5%). The frequentist analysis conducted by Pearse et al. [12] identified no significant difference for 30-day complications/death between the treatment arms and deemed the trial to be underpowered. The Bayesian posterior mean estimates and 95% HDI based on the flat priors gave similar results to the frequentist analysis as expected, with more optimistic results produced by the evidence-based prior. (Previous evidence had suggested that the intervention was superior.) The primary advantage here of using the Bayesian approach is that we can explicitly obtain the posterior distribution of the primary outcome rates for each arm, and from this the treatment effect distribution, which can be interpreted directly and intuitively. From these distributions we can calculate the probability that the new treatment is superior. A frequentist analysis, particularly of an underpowered trial, provides little help for clinicians to make the necessary decisions regarding future treatment and also runs the risk of concluding no difference between treatments (based on a non-significant p-value), even when there is in reality a useful difference. By incorporating previous information into the prior distribution, the Bayesian approach can provide more informative probabilistic statements about the treatment effect.

For a reasonably large trial, different priors should have little effect on the posterior distributions as the data are contributing a large amount of information, and generally outweigh the contribution of the prior information. In this study, altering the priors had little impact on the results, most likely due to the fairly large sample size. The evidence-based prior used information from 21 small studies whose sample sizes ranged from 34 to 390 patients and were mostly in favour of the intervention. The evidence-based prior for the control arm contributed 56 patients worth of information compared to the 364 patients from OPTIMISE; the evidence-based prior for the intervention arm contributed 66 patients worth of information and the OPTIMISE trial contributed 366 patients. The analyses which used the evidence-based priors obtained a slightly smaller RR and provided more certainty that the intervention was superior, compared to the flat priors. Since the previous studies were small studies, one may wish to down weight the influence of these studies in the prior to consider the risk of bias. Plots of the priors overlaid with the posteriors for the evidence-based priors

For peer review only

are provided in the Supplementary Material (Figure S3) and show that the prior for the intervention was too optimistic. This is likely due to the small size of many of the previous studies and the large degree of heterogeneity between studies. Publication bias may also be present.

The Bayesian analyses that we have presented here are simple, and repeat the primary analysis of the OPTIMISE trial to demonstrate the usefulness of the Bayesian approach. These analyses could be extended to allow for more complicated models, such as Bayesian hierarchical models that account for site-specific effects or to allow for covariate adjustment. A Bayesian analysis cannot salvage a badly designed or conducted trial. Instead, the Bayesian approach can be used to produce results that are more easily interpreted and avoids the dichotomisations that occur in significance testing.

Conclusion

Using the results from a standard statistical analysis, the OPTIMISE trial authors concluded that the use of the intervention compared with usual care did not significantly reduce a composite outcome of 30-day complications and mortality [12]. Although the trial was not powered to detect a statistically significant difference between the treatment arms for the observed effect size, by using Bayesian analyses we were able to demonstrate that the intervention was superior to the control with 96.9-99.5% probability. These results are much more clinically useful than those that are given by the traditional approach.

Figure legends:

Figure 1. Prior distributions used in Bayesian reanalysis of OPTIMISE. The top panel is a flat prior distribution, beta(1,1), that was used for both treatment arms. The middle and bottom panels show the evidence-based priors that were derived using a meta-analysis. The middle panel is the prior for the control arm (beta(24.26, 31.58)) and the bottom panel is the prior for the intervention arm (beta(16.46, 49.17)).

Figure 2. Posterior distributions of relative risk (RR) and absolute risk difference for the flat prior and evidence-based priors. $\theta 1$ and $\theta 2$ are the composite outcome rates in the intervention and control arms, respectively. Posterior distribution of the RR of the primary outcome using a) a flat prior, b) evidence-based priors. A RR>1 indicates that the intervention is more harmful. Posterior distribution of the absolute risk difference in the primary outcome using c) a flat prior d) evidence-based priors. A positive value of the absolute risk difference indicates that the intervention is more harmful. The 95% highest posterior density interval (HDI) is shown as the thick black horizontal line with the boundaries written above the line. The region of practical equivalence (ROPE) is shown in red (with dotted vertical lines). The posterior probability of RR<1 and RR>1, and an absolute difference <0 and >0 is shown in green above the ROPE

Figure 3. Posterior probability of specified effect sizes, using (a) flat prior, (b) evidence-based priors. The line shows the probability of the RR being lower than the values on the x-axis (i.e., a bigger treatment effect). A RR<1 indicates that the primary outcome rate is smaller in the intervention arm compared to the control arm

References

For peer review only

[1] Simmons JP, Nelson LD, Simonsohn U. False-positive psychology: Undisclosed flexibility in data collection and analysis allows presenting anything as significant. Psychol Sci. 2011 Oct 17; 22(11):1359-1366.

[2] Greenland S, Senn SJ, Rothman KJ, Carlin JB, Poole CP, Goodman SN, Altman DG. Statistical tests, *P* values, confidence intervals, and power: a guide to misinterpretations. Eur J Epidemiol. 2016 May 21; 31: 337-350.

[3] Wasserstein RL, Lazar NA. The ASA's statement on p-values: context, process and purpose. The American Statistician. 2016 June 09; 70(2): 129-133

[4] McShane BB, Gal D, Gelman A, Robert C, Tackett JL. Abandon Statistical Significance. arXiv preprint arXiv:1709.07588. 2017 Sep 21.

[5] Alderson P, Chalmers I. Survey of claims of no effect in abstracts of Cochrane reviews. BMJ. 2003 Mar 01; 326(7387):475.

[6] Greenland S. Null misinterpretation in statistical testing and its impact on health risk assessment. Prev Med. 2011 Aug 17; 53:225–8.

[7] Altman DG, Bland JM. Statistics notes: Absence of evidence is not evidence of absence. BMJ. 1995 Aug 19; 311(7003):485.

[8] Trafimow D, Marks M. Editorial. Basic Appl. Soc. Psych. 2015 Feb 12; 37(1): 1-2

[9] Morris RK, Malin GL, Quinlan-Jones E, Middleton LJ, Hemming K, Burke D, et al. Percutaneous vesicoamniotic shunting versus conservative management for fetal lower urinary tract obstruction (PLUTO): a randomised trial. Lancet. 2013 Aug 13; 382(9903): 1496-1506.

[10] Laptook AR, Shankaran S, Tyson JE, Munoz B, Bell EF, Goldberg RN, et al. Effect of Therapeutic Hypothermia Initiated After 6 Hours of Age on Death or Disability Among Newborns With Hypoxic-Ischemic Encephalopathy A Randomized Clinical Trial. JAMA. 2017 October 31; 318(16): 1550-1560.

[11] Reardon MJ, Van Miegham NM, Popma JJ, Kleiman NS, Sondergaard L, Mumtaz M, et al. Surgical or Transcatheter Aortic-Valve Replacement in Intermediate-Risk Patients. NEJM. 2017 Mar 17; 376(14): 1321-1331.

[12] Pearse RM, Harrison DA, MacDonald N, Gillies MA, Blunt M, Ackland G, et al. Effect of a Perioperative, Cardiac Output–Guided Hemodynamic Therapy Algorithm on Outcomes Following Major Gastrointestinal Surgery A Randomized Clinical Trial and Systematic Review. JAMA. 2014 May 19; 311(21): 2181-2190.

[13] Grocott MP, Dushianthan A, Hamilton MA, Mythen MG, Harrison D, Rowan K; Optimisation Systematic Review Steering Group. Perioperative increase in global blood flow to explicit defined goals and outcomes following surgery. *Cochrane Database Syst Rev*. 2012 Nov 14;11:CD004082.

For peer review only

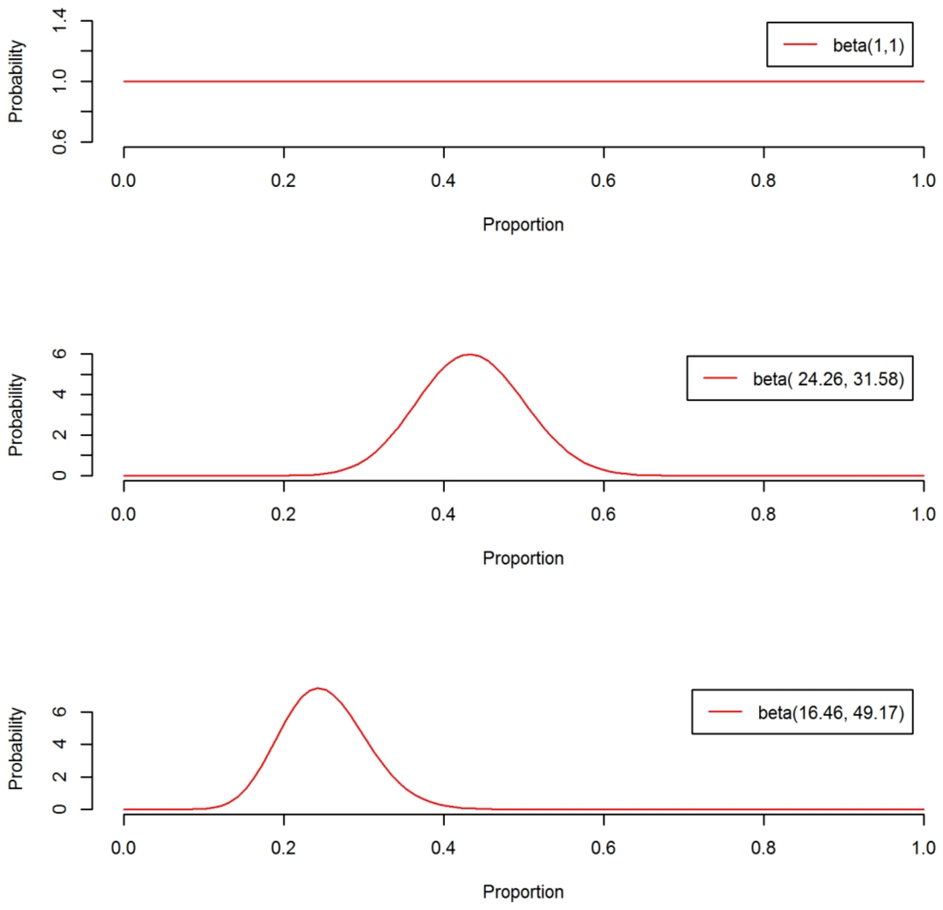

Figure 1. Prior distributions used in Bayesian reanalysis of OPTIMISE. The top panel is a flat prior distribution, beta(1,1), that was used for both treatment arms. The middle and bottom panels show the evidence-based priors that were derived using a meta-analysis. The middle panel is the prior for the control arm (beta(24.26, 31.58)) and the bottom panel is the prior for the intervention arm (beta(16.46, 49.17)).

152x152mm (300 x 300 DPI)

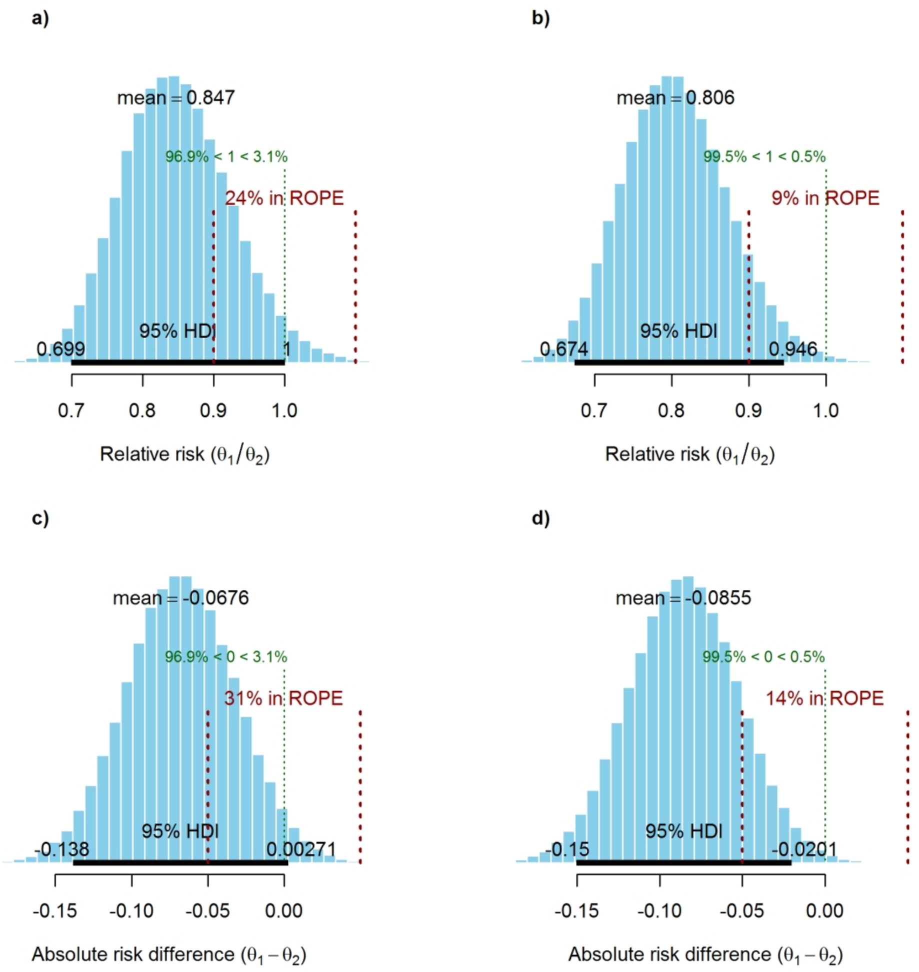

Figure 2. Posterior distributions of relative risk (RR) and absolute risk difference for the flat prior and evidence-based priors. θ1 and θ2 are the composite outcome rates in the intervention and control arms, respectively. Posterior distribution of the RR of the primary outcome using a) a flat prior, b) evidence-based priors. A RR>1 indicates that the intervention is more harmful. Posterior distribution of the absolute risk difference in the primary outcome using c) a flat prior d) evidence-based priors. A positive value of the absolute risk difference indicates that the intervention is more harmful. The 95% highest posterior density interval (HDI) is shown as the thick black horizontal line with the boundaries written above the line. The region of practical equivalence (ROPE) is shown in red (with dotted vertical lines). The posterior probability of RR<1 and RR>1, and an absolute difference <0 and >0 is shown in green above the ROPE

177x177mm (300 x 300 DPI)

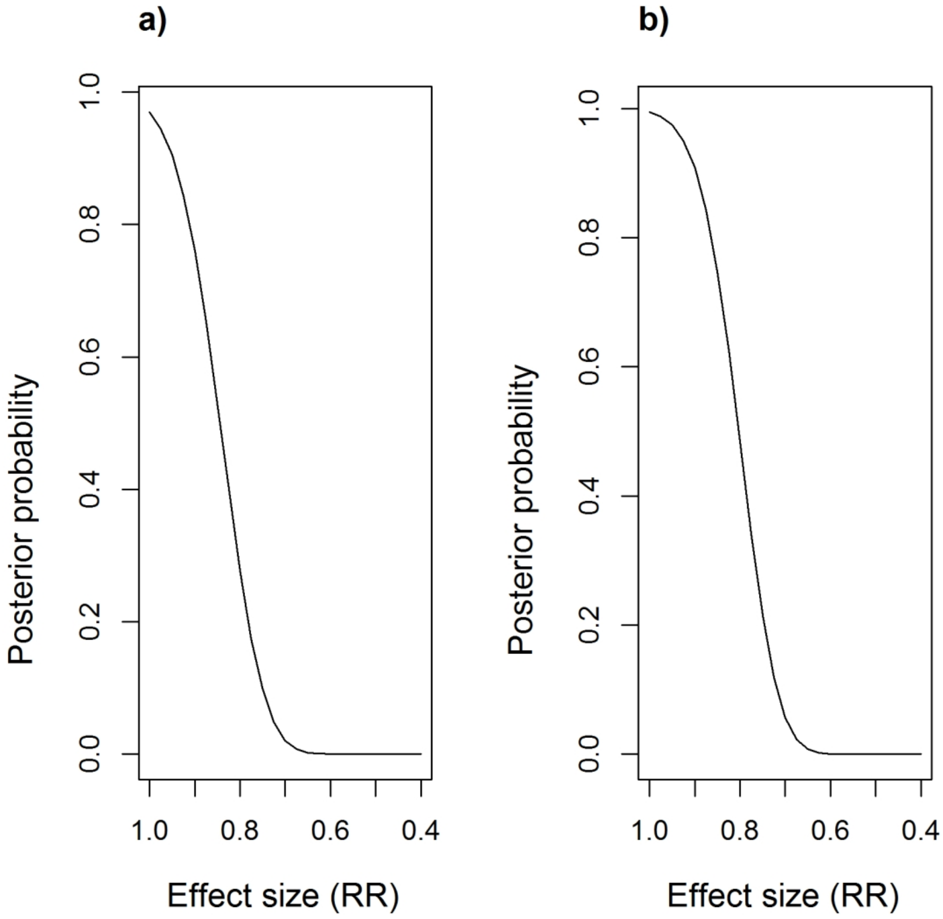

Figure 3. Posterior probability of specified effect sizes, using (a) flat prior, (b) evidence-based priors. The line shows the probability of the RR being lower than the values on the x-axis (i.e., a bigger treatment effect). A RR<1 indicates that the primary outcome rate is smaller in the intervention arm compared to the control arm

152x152mm (300 x 300 DPI)

Supplementary Material

Algorithms settings and code for Bayesian analysis

The Bayesian analysis was conducted in R version 3.4.1 using the *rjags* package [1], which provides an interface to the JAGS library (JAGS version 4.3.0) to perform Bayesian data analysis. JAGS (Just Another Gibbs Sampler) was derived from BUGS (Bayesian analysis Using Gibbs Sampling; see [2] for details on the BUGS project) and uses Markov Chain Monte Carlo (MCMC) to generate samples from the posterior distribution so that inference on the parameters of interest may be obtained. The posterior distribution was obtained using a sample of 200 000 estimates of the primary outcome probability for each arm (used 3 chains each of length 66667, with a burn-in period of 1000 iterations and 500 iterations were used to adapt/tune the samplers). Model convergence was checked using trace plots, density plots, and the Gelman-Rubin diagnostic [3]. The MCMC code is presented below in “R code 1”.

Since we used Beta distributions for the priors, and have binary data that is modelled by a binomial distribution, we are able to obtain an algebraic expression to calculate the posterior distribution. The Beta distribution prior is a *conjugate prior* for the binomial distribution, which means that the posterior distribution is in the same probability distribution family as the prior distribution (Beta distribution). We can update the Beta distribution parameters using the data from the trial:

$$Beta(\alpha_t + y_t, \beta_t + N_t - y_t)$$

where $\mathrm{Beta}(\alpha_{t,}, \beta_t)$ is the prior for treatment arm *t; y_t* are the number of complications/deaths at 30 days for arm t; and N_t are the number of patients randomised to arm t. We can simulate samples from this distribution for each arm (simulating from the posterior) and examine the relative risks (RR) and absolute risk differences. This code is presented in “R code 2”.

R code 1. Code for posterior simulations using an MCMC algorithm

```
# OPTIMISE trial in a Bayesian framework

# JAMA. 2014;311(21):2181-2190. doi:10.1001/jama.2014.5305


remove(list=ls())


#specify your working directory

setwd("H:/Documents/optimise i")




# Primary outcome: composite of 30-day moderate or major complications and mortality

N1 <- 366

y1 <- 134
```

For peer review only - http://bmjopen.bmj.com/site/about/guidelines.xhtml

```
N2 <- 364

y2 <- 158

# N1 is total number in the Cardiac Output–Guided Hemodynamic Therapy Algorithm (intervention) group

# y1 is number with the outcome in the Cardiac Output–Guided Hemodynamic Therapy Algorithm (intervention) group

# N2 is total number in usual care (control) group

# y2 is number with the outcome in usual care (control) group


# Risk ratio

(y1/N1)/(y2/N2)


#frequentist analysis

library(epitools)

riskratio(c(N1-y1, y1, N2-y2, y2), rev="rows", method="boot", replicates=100000)


# Using standard frequentist approach

# Risk ratio (bootstrapped 95% confidence intervals) = 0.84 (0.70-1.01)

# p=0.07 (Fisher exact p-value)



#-----------------------------------------------------------------------------


# Bayesian analysis of same ratio

# Base script from Kruschke, J. K. (2011). Doing Bayesian Data Analysis, Academic Press / Elsevier.


#load required packages (install first if necessary)

require(rjags)

library(BEST)



# The model will be specified with uninformative prior distributions (beta(1,1,).


# Plot prior beta(1,1)

par(mfrow=c(1,1))

plot(seq(0, 1, length.out=100), dbeta(seq(0, 1, length.out=100), 1,1),

                    type="l", xlab="Proportion",

                    ylab="Probability",
```

For peer review only

```
                    main="OPTIMISE Composite Primary Outcome Prior distribution",
                    frame=FALSE, col="red", oma=c(6,6,6,6))
legend("topright", legend="beta(1,1)", lty=1, col="red", inset=0)


#------------------------------------------------------------------------------


# Specify the MODEL.
modelString = "
# JAGS model specification begins here...
model {
# Likelihood. Each complication/death is Bernoulli.
for ( i in 1 : N1 ) { y1[i] ~ dbern( theta1 ) }
for ( i in 1 : N2 ) { y2[i] ~ dbern( theta2 ) }
# Prior. Independent beta distributions. Change this for informative prior
theta1 ~ dbeta( 1,1) #intervention
theta2 ~ dbeta( 1,1) #control
}
# ... end JAGS model specification
" # close quote for modelstring

# Write the modelString to a file, using R commands:
writeLines(modelString,con="H:/Documents/optimise i/model.txt")



#------------------------------------------------------------------------------
# THE DATA.

# Specify the data in a form that is compatible with JAGS model, as a list:
dataList =          list(
         N1 = N1 ,
         y1 = c(rep(1, y1), rep(0, N1-y1)),
         N2 = N2 ,
         y2 = c(rep(1, y2), rep(0, N2-y2))
)

#------------------------------------------------------------------------------
```

For peer review only

```
# INTIALIZE THE CHAIN.

# Can be done automatically in jags.model() by commenting out inits argument.
# Otherwise could be established as:
# initsList = list( theta1 = sum(dataList$y1)/length(dataList$y1) ,
#                   theta2 = sum(dataList$y2)/length(dataList$y2) )

#------------------------------------------------------------------------------
# RUN THE CHAINS.

#specify the MCMC parameters
parameters = c( "theta1" , "theta2" )     # The parameter(s) to be monitored.
adaptSteps = 500              # Number of steps to "tune" the samplers.
burnInSteps = 1000            # Number of steps to "burn-in" the samplers.
nChains = 3                   # Number of chains to run.
numSavedSteps=200000          # Total number of steps in chains to save.
thinSteps=1                   # Number of steps to "thin" (1=keep every step).
nIter = ceiling( ( numSavedSteps * thinSteps ) / nChains ) # Steps per chain.



# Create, initialize, and adapt the model:
jagsModel = jags.model( "H:/Documents/optimise i/model.txt" , data=dataList , # inits=initsList ,
                        n.chains=nChains , n.adapt=adaptSteps )
# Burn-in:
cat( "Burning in the MCMC chain...n" )

#run the MCMC
update( jagsModel , n.iter=burnInSteps )

# The saved MCMC chain:
cat( "Sampling final MCMC chain...n" )
codaSamples = coda.samples( jagsModel , variable.names=parameters ,
                        n.iter=nIter , thin=thinSteps )

# resulting codaSamples object has these indices:
```

For peer review only

```
#   codaSamples[[ chainIdx ]][ stepIdx , paramIdx ]



#check MCMC has converged

plot(codaSamples)

summary(window(codaSamples, start = burnInSteps))

# Gelman-Rubin diagostic - factor of 1 means that between chain variance and within chain variance are equal. Values <=1.1 suggest convergence

gelman.diag(codaSamples)

#Check whether the low chain reduction is stable.

gelman.plot(codaSamples)

#Burn-in appear sufficient (choose the iteration after which the lines stay below 1.1)



#------------------------------------------------------------------------------

# EXAMINE THE RESULTS.


# Convert coda-object codaSamples to matrix object for easier handling.

# This concatenates the different chains into one long chain.

# Result is mcmcChain[ stepIdx , paramIdx ]

mcmcChain = as.matrix( codaSamples )



theta1Sample = mcmcChain[,"theta1"] # Put sampled values in a vector - INTERVENTION ARM.

theta2Sample = mcmcChain[,"theta2"] # Put sampled values in a vector - CONTROL ARM




# Plot a histogram of the posterior differences of theta values.

thetaRR = theta1Sample / theta2Sample # Relative risk

thetaDiff = theta1Sample - theta2Sample # Absolute risk difference


# 2x1 plots

par(mfrow=c(2,1))


# plot posterior of RR

par(mar=c(4, 4.1, 4.1, 4))
```

For peer review only

```
plotPost( thetaRR , xlab= expression(paste("Relative risk (", theta[1]/theta[2], ")")) ,
        compVal=1.0, ROPE=c(0.9, 1.1),
        main="OPTIMISE Composite Primary Outcome
Posterior distribution of relative risk", cex.main=1, cex=1, cex.lab=1, xlab=c(0.6,1.4))

#Plot absolute risk difference
plotPost( thetaDiff , xlab=expression(paste("Absolute risk difference (", theta[1]-theta[2], ")")) ,
        compVal=0.0, ROPE=c(-0.05, 0.05),
        main="OPTIMISE Composite Primary Outcome
Posterior distribution of absolute risk difference", cex.main=1, cex=1, cex.lab=1, xlab=c(-0.25, 0.15))




#-----------------------------------------------------------------------------

# Use posterior prediction to determine proportion of cases in which
# using the intervention would result in no complication/death
# while not using the intervention would result in complication death

chainLength = length( theta1Sample )

# Create matrix to hold results of simulated patients:
yPred = matrix( NA , nrow=2 , ncol=chainLength )

# For each step in chain, use posterior prediction to determine outcome
for ( stepIdx in 1:chainLength ) { # step through the chain
        # Probability for complication/death for each "patient" in intervention group:
        pDeath1 = theta1Sample[stepIdx]
        # Simulated outcome for each intervention "patient"
        yPred[1,stepIdx] = sample( x=c(0,1), prob=c(1-pDeath1,pDeath1), size=1 )
        # Probability for complication/death for each "patient" in control group:
        pDeath2 = theta2Sample[stepIdx]
        # Simulated outcome for each control "patient"
        yPred[2,stepIdx] = sample( x=c(0,1), prob=c(1-pDeath2,pDeath2), size=1 )
}
```

For peer review only

```
# Now determine the proportion of times that the intervention group has no complication/death
# (y1 == 0) and the control group does have a complication or death (y2 == 1))
(pY1eq0andY2eq1 = sum( yPred[1,]==0 & yPred[2,]==1 ) / chainLength)


#proportion of times intervention has complication/death where control group does not
(pY1eq1andY2eq0 = sum( yPred[1,]==1 & yPred[2,]==0 ) / chainLength)


#proportion of times where neither arm has complication/death
(pY1eq0andY2eq0 = sum( yPred[1,]==0 & yPred[2,]==0 ) / chainLength)


#proportion of times where both arms have complication/death
(pY10eq1andY2eq1 = sum( yPred[1,]==1 & yPred[2,]==1 ) / chainLength)
```

For peer review only

R Code 2. Code for exact posterior calculation

```
remove(list=ls())

###uninformative prior

#prior for control group is beta(a1, b1)
a1=1
b1=1

#prior for treatment group is beta(a2, b2)
a2=1
b2=1

#control event/total is z1/N1
z1=158
N1=364

#treatment event/total is z2/N2.
z2=134
N2=366

#simulate from the posterior for control group. posterior can be calculated exactly as beta(a1+z1, b1+N1-z1)
pc = rbeta(200000, a1+z1, b1+N1-z1)
#simulate from the posterior for intervention group. posterior can be calculated exactly as beta(a2+z2, b2+N2-z2)
pt = rbeta(200000, a2+z2, b2+N2-z2)


thetaRR = pt / pc # Relative risk
thetaDiff = pt - pc # Absolute risk difference

##########################################################################
##########################################################################
#evidence-based prior

#prior for control group is beta(a1, b1)
a1=24.26
b1=31.58


#prior for treatment group is beta(a2, b2)
a2=16.46
b2=49.17

#control event/total is z1/N1
z1=158
N1=364

#treatment event/total is z2/N2.
z2=134
N2=366

#simulate from the posterior for control group. posterior can be calculated exactly as beta(a1+z1, b1+N1-z1)
```

For peer review only

```
pcc = rbeta(200000, a1+z1, b1+N1-z1)
#simulate from the posterior for intervention group. posterior can be calculated exactly as beta(a2+z2, b2+N2-z2)
ptt = rbeta(200000, a2+z2, b2+N2-z2)



thetaRR_ev = ptt / pcc # Relative risk
thetaDiff_ev = ptt - pcc # Absolute risk difference


##########################################################################

#plot posterior distributions

require(rjags)
library(BEST)

# 2x2 plots
par(mfrow=c(2,2))

# plot posterior for uninformative prior, RR
par(mar=c(4, 4.1, 4.1, 4))
plotPost( thetaRR , xlab= expression(paste("        Relative risk (", theta[1]/theta[2], ")")) ,
          compVal=1.0, ROPE=c(0.9, 1.1),
          main="a)", adj=0,cex.main=1, cex=1, cex.lab=1, xlab=c(0.6,1.4))

# plot posterior for uninformative prior, absolute risk difference
plotPost( thetaDiff , xlab=expression(paste("Absolute risk difference (", theta[1]-theta[2], ")")) ,
          compVal=0.0, ROPE=c(-0.05, 0.05),
          main="b)", adj=0,cex.main=1, cex=1, cex.lab=1, xlab=c(-0.25, 0.15))

# plot posterior for evidence-based prior, RR
par(mar=c(4, 4.1, 4.1, 4))
plotPost( thetaRR_ev , xlab= expression(paste("        Relative risk (", theta[1]/theta[2], ")")) ,
          compVal=1.0, ROPE=c(0.9, 1.1),
          main="c)", adj=0,cex.main=1, cex=1, cex.lab=1, xlab=c(0.6,1.4))

# plot posterior for evidence-based prior, absolute risk difference
plotPost( thetaDiff_ev , xlab=expression(paste("Absolute risk difference (", theta[1]-theta[2], ")")) ,
          compVal=0.0, ROPE=c(-0.05, 0.05),
          main="d)", adj=0,cex.main=1, cex=1, cex.lab=1, xlab=c(-0.25, 0.15))
```

For peer review only

Evidence-based Prior

An evidence-based prior was specified using results from a pre-existing meta-analysis ([4,5] – not including the OPTIMISE results) which had information on both treatment arms. There is no clear best approach of how to include historical information into the analysis of a clinical trial. Viele et al. [6] provide a good overview of the available approaches when data for the control arm is available and discuss how to decide to what extent historical data should be incorporated.

The R Bayesian evidence synthesis Tools (RBesT; [7]) were used to perform a Bayesian meta-analysis, which accounts for the uncertainty of the population mean and between-trial heterogeneity (using the *gMAP* function in R (version 3.4.1)). We used this approach as it is a principled and reproducible method of combining data from previous trials. Further refinements could also be included, such as attempting to correct for publication or other biases. The meta-analysis of the control and intervention arms are displayed in Figures S1 and S2, respectively.

For the control arm, the meta-analysis gave a mean estimate of 0.432 (95% HDI: 0.306, 0.563). Using the *betaExpert* function (from the *prevalence* package [8]) in R version 3.4.1 (setting the mean from the meta-analysis to the mode of the beta distribution, and using the 95% HDI) gave a beta(24.26,31.58) distribution. This was used as the prior for the control arm.

For the intervention arm, the meta-analysis gave a mean estimate of 0.243 (95% HDI: 0.148, 0.358). Using the *betaExpert* function [8] in R (setting the mean from the meta-analysis to the mode of the beta distribution, and using the 95% HDI) gave a beta(16.46, 49.17) distribution. This was used as the prior for the intervention arm.

We note that power priors [9] could have been used instead for deriving the evidence-based priors, which incorporate a weight parameter that determines how much information is used from the historical data.

For peer review only

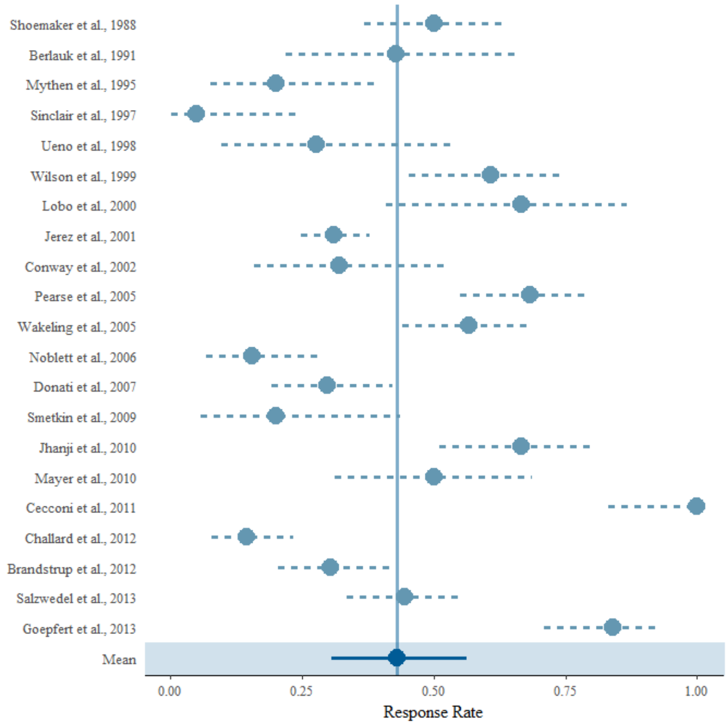

Figure S1.Forest plot of control arm using meta-analysis from Pearse et al. [5]. Dashed lines represent the stratified estimates with 95% HDI. Solid line represents the mean estimate (with 95% HDI))

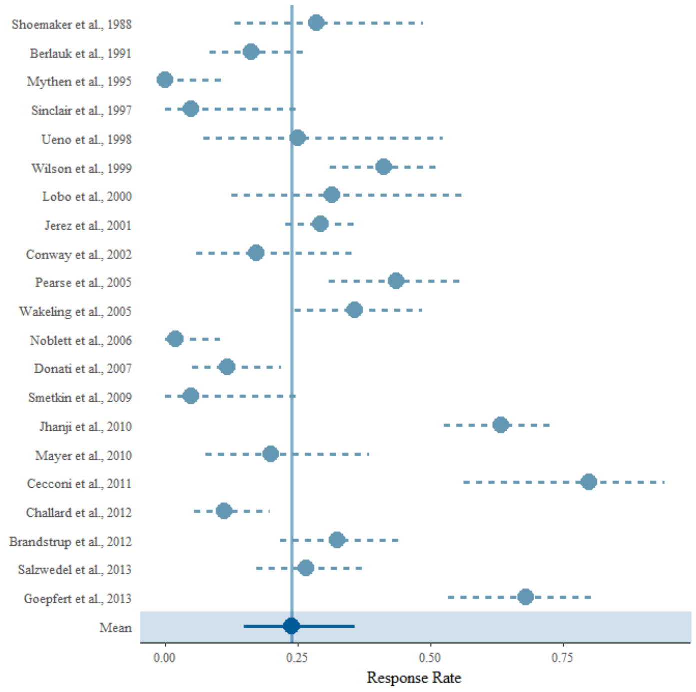

Figure S2. Forest plot of intervention arm using meta-analysis from Pearse et al. [5]. Dashed lines represent the stratified estimates with 95% HDI. Solid line represents the mean estimate (with 95% HDI))

For peer review only

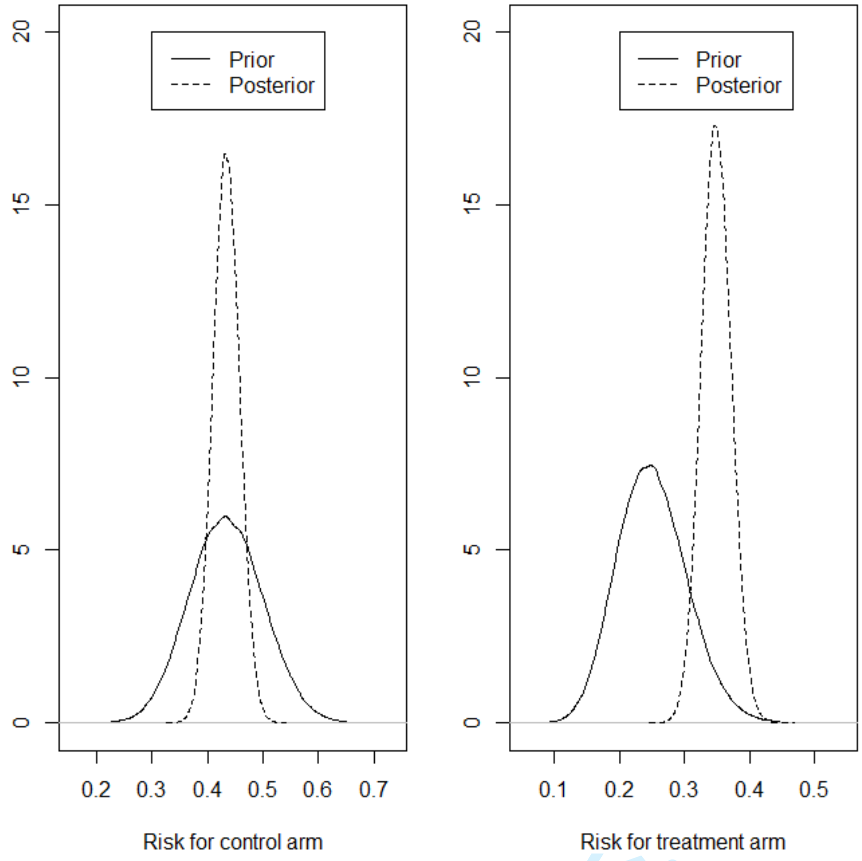

Figure S3. Plot of the evidence-based priors overlaid with the posteriors for each treatment arm

References

[1] Plummer M. JAGS: A program for analysis of Bayesian graphical models using Gibbs sampling. Proceedings of the 3rd International Workshop on Distributed Statistical Computing (DSC 2003). March 2003. p. 20–2.

[2] Lunn D, Spiegelhalter D, Thomas A, Best N. The BUGS project: Evolution, critique and future directions. Stat Med. 2009 October 13; 28(25): 3049-3067

[3] Gelman A, Rubin DB. Inference from Iterative Simulation Using Multiple Sequences. Stat Sci. 1992; 7(4): 457-472.

[4] Grocott MP, Dushianthan A, Hamilton MA, Mythen MG, Harrison D, Rowan K; Optimisation Systematic Review Steering Group. Perioperative increase in global blood flow to explicit defined goals and outcomes following surgery. *Cochrane Database Syst Rev*. 2012 Nov 14;11:CD004082.

[5] Pearse RM, Harrison DA, MacDonald N, Gillies MA, Blunt M, Ackland G, et al. Effect of a Perioperative, Cardiac Output–Guided Hemodynamic Therapy Algorithm on Outcomes

For peer review only - http://bmjopen.bmj.com/site/about/guidelines.xhtml

Following Major Gastrointestinal Surgery A Randomized Clinical Trial and Systematic Review. JAMA. 2014 May 19; 311(21): 2181-2190.

[6] Viele K, Berry S, Neuenschwander B, Amzal B, Chen F, Enas N, et al. Use of historical control data for assessing treatment effects in clinical trials. Pharm Stat. 2013 August 5; 13:41-54

[7] Weber S. (2017). RBesT: R Bayesian Evidence Synthesis Tools. R package version 1.2-3. https://CRAN.R-project.org/package=RBesT

[8] Devleesschauwer B, Torgerson P, Charlier J, Levecke B, Praet N, Roelandt S, et al. (2014). prevalence: Tools for prevalence assessment studies. R package version 0.4.0. http://cran.r-project.org/package=prevalence

[9] Chen M-H, Ibrahim JG, Shao Q-M. Power prior distributions for generalized linear models. J Stat Plan Infer. 2000 Mar; 84(1-2):121-137

For peer review only
